# Overview on Multienzymatic Cascades for the Production of Non-canonical α-Amino Acids

**DOI:** 10.3389/fbioe.2020.00887

**Published:** 2020-08-11

**Authors:** Sergio Martínez-Rodríguez, Jesus M. Torres, Pilar Sánchez, Esperanza Ortega

**Affiliations:** Department of Biochemistry and Molecular Biology III and Immunology, University of Granada, Granada, Spain

**Keywords:** amino acid, non-canonical, enzyme, cascade, industrial process, proteinogenic, biotechnology

## Abstract

The 22 genetically encoded amino acids (AAs) present in proteins (the 20 standard AAs together with selenocysteine and pyrrolysine), are commonly referred as proteinogenic AAs in the literature due to their appearance in ribosome-synthetized polypeptides. Beyond the borders of this key set of compounds, the rest of AAs are generally named imprecisely as non-proteinogenic AAs, even when they can also appear in polypeptide chains as a result of post-transductional machinery. Besides their importance as metabolites in life, many of D-α- and L-α-“non-canonical” amino acids (NcAAs) are of interest in the biotechnological and biomedical fields. They have found numerous applications in the discovery of new medicines and antibiotics, drug synthesis, cosmetic, and nutritional compounds, or in the improvement of protein and peptide pharmaceuticals. In addition to the numerous studies dealing with the asymmetric synthesis of NcAAs, many different enzymatic pathways have been reported in the literature allowing for the biosynthesis of NcAAs. Due to the huge heterogeneity of this group of molecules, this review is devoted to provide an overview on different established multienzymatic cascades for the production of non-canonical D-α- and L-α-AAs, supplying neophyte and experienced professionals in this field with different illustrative examples in the literature. Whereas the discovery of new or newly designed enzymes is of great interest, dusting off previous enzymatic methodologies by a “*back and to the future*” strategy might accelerate the implementation of new or improved multienzymatic cascades.

## Introduction

Chemically, an amino acid (AA) is any molecule containing a carboxylic acid and an amino group. This family of compounds is hugely heterogeneous, and includes any linear or cyclic molecule containing both substituents. The amino moiety can be situated at the α-, β-, γ-, δ-, etc. position with respect to the carbonyl group of the acid. AAs can be further functionalized with other substituents, additionally heterogenizing this family of compounds. Nonetheless, the L-isomer of α-AAs occupies a central role in biosciences, since these molecules were evolutionarily chosen to become the building blocks of one of the macromolecular scaffolds sustaining life. The 22 genetically encoded AAs present in proteins (the 20 standard AAs together with selenocysteine and pyrrolysine) are commonly referred as proteinogenic amino acids (PAAs). On the other hand, the IUPAC recommended the terminology “common amino acids” for genetically-encoded AAs and other AAs with known biological functions, such as L-DOPA (IUPAC, and IUb, 1984). By the end of the seventies, more than 140 natural AAs and AA-derivatives had been proposed in natural proteins (Uy and Wold, 1977; Hunt, 1985); more than 900 NcAAs have been suggested in plants (Yamane et al., 2010). Thousands of synthetic NcAAs not occurring naturally have been chemically synthesized by different approaches (Xue et al., 2018). In this sense, the “proteinogenic” or “non-proteinogenic” classification of AAs can result imprecise, as non-coded AAs are also commonly found in significant quantities in proteins, such as hydroxylysine and hydroxyproline. Several D-α-AAs are also widely described in peptides and proteins (Martínez-Rodríguez et al., 2010c; Grishin et al., 2020), and the emerging role of its metabolism in the innate defense has been recently suggested (Sasabe and Suzuki, 2018). On the other hand, AAs not included in the above-mentioned group have also been referred to as non-protein, non-natural, unnatural, non-canonical, non-proteinogenic or unusual, among other terminologies (Hunt, 1985; Fan et al., 2015; Blaskovich, 2016; Agostini et al., 2017; Baumann et al., 2017). We will refer to them as “non-canonical amino acids” (NcAAs).

The occurrence and biological activity of NcAAs *in vivo* is hugely diverse, and some of their properties have been reflected in different papers (Wagner and Musso, 1983; Hunt, 1985; Nunn et al., 2010; Yamane et al., 2010; Walsh et al., 2013; Blaskovich, 2016; Zou et al., 2018; Narancic et al., 2019; Hedges and Ryan, 2020). Broadly, several NcAAs are utilized as intermediates in primary metabolic pathways (e.g., homoserine, ornithine, citrulline,…). Free NcAAs also possess antimicrobial, antiproliferative, anti-inflammatory, or other biologically relevant activity (Martínez-Rodríguez et al., 2010c; Blaskovich, 2016). They serve as building blocks for many different small bioactive peptide scaffolds (Walsh et al., 2013), including hormones (Martínez-Rodríguez et al., 2010c), or confer specific structural properties to proteins (i.e., collagen or insulin). The activity of many NcAAs have been proposed not directly related with the physiology of the organism itself, but with its relationship with other organisms in its environment, suggesting physiological (toxic), deterrent (pheromonal) or other modifying roles external to the responsible species (Hunt, 1985). Microorganisms are a clear example on how evolution has taken advantage of using NcAAs over competing organisms (Nunn et al., 2010), and microbial antibiotics have been widely described (Martínez-Rodríguez et al., 2010c; Walsh et al., 2013; Blaskovich, 2016). Many of the isolated NcAAs or peptide-containing NcAAs also serve as plant-defense against predators, pathogens or other organisms competing for the same resources (Yamane et al., 2010; Rodgers, 2014). Furthermore, some NcAAs have also been shown to be toxic for humans and domestic animals (Bell, 2003; Nunn et al., 2010; Rodgers, 2014), and have been suggested as potentially linked to neurodegenerative diseases (Rodgers, 2014).

### Biomedical and Biotechnological Applications of NcAAs

Enantiopure NcAAs are of considerable economic importance because of their broad industrial applications (Patel, 2013; Narancic et al., 2019). The global AA market increased from 0.7 million tons in 1985 to 9.3 Million Tons in 2019, and is expected to reach a volume of 11.9 million tons in 2025 (Ikeda and Takeno, 2013; IMARC group, 2019). A “Compound Annual Growth Rate” (CAGR) of 5.6% is expected from 2015 to 2022 (Sánchez et al., 2018). Amino acid global sales reached $20 billion in 2014 and are predicted to exceed $35 billion by 2022 (Yan and Wang, 2019). Besides the advances focused on the million-ton scale bulk PAAs L-glutamate and L-lysine, the Industry continues widening its interest toward other specialty NcAAs (Wendisch, 2020), and there is strong commercial interest in developing new amino acid applications (Sánchez et al., 2018). In fact, some NcAAs have proven key for the production of many of the 200 top-grossing pharmaceuticals (Boville et al., 2018b). Some examples are sitagliptin (Merck, $5.91 billion), pregabalin (Pfizer, $4.62 billion) or glecaprevir (AbbVie, $3.44 billion) (Belk, 2018).

From the biotechnological and biomedical point of view, NcAAs have found applications as a significant expansion of the building-block repertoire and/or as organocatalysts (Agirre et al., 2019), also in the manufacture of a wide range of pharmaceuticals (Patel, 2013; Narancic et al., 2019), or as linear and cyclic peptides (Blaskovich, 2016; Martin et al., 2018). Some of the commercial applications directly rely on the natural properties of peptide-containing NcAAs, such as several antibiotics. Norine, an online database, contains updated and important information on different NcAAs present in non-ribosomal peptides (Flissi et al., 2020). Besides the direct commercial applications of NcAAs or derived NcAA-compounds, they have also been used into structure-activity relationships (SAR) peptides (Blaskovich, 2016); incorporation of NcAAs into protein and peptides using ribosomes is an increasing field with many possibilities (Martin et al., 2018). *In vivo* incorporation of NcAAs into antimicrobial peptides is also an enlarging field of study due to the potential discovery of novel antibiotics to broaden the human pharmacological barriers toward microbes. Genetic engineering methodologies allow gene libraries of 10^1^ –10^8^ variants and thus, random incorporation of NcAAs in their sequence; sampling can thus be carried out by high throughput approaches, greatly shortening the time needed for new antimicrobial development (Baumann et al., 2017). Among them, the possibility to engineer proteins to incorporate site-specifically different NcAAs has had a major impact the development of this field (Agostini et al., 2017; Zhao et al., 2020). This technology has removed the constraints imposed by nature on the use of the 22-genetically encoded AAs, allowing to insert new properties and/or improved functionalities on protein scaffolds. Some examples are improvements in enzyme catalytic efficiency, chemical and/or thermal stability, substrate scope, enantio- and stereoselectivity or inhibition. Also, bioorthogonal functionalization, spectroscopic probes, photo-cross-linking, metal-chelating or post-translational modification-mimicking has been reported (Agostini et al., 2017; Almhjell and Mills, 2018; Rezhdo et al., 2019; Zhao et al., 2020). Thus, the huge advances on the standardization of methodologies for the production of enzymes incorportating NcAAs (Smolskaya and Andreev, 2019; Hammerling et al., 2020) are also boosting the application and economic significance of NcAAs.

Among the numerous L-α-NcAAs with biomedical interest (Table 1), L-homophenylalanine is a precursor for the preparation of valuable angiotensin-converting enzyme (ACE) and renin inhibitors (e.g., enalapril, lisinopril, quinapril, ramipril, trandolapril or benazepril, among others; Ahmad et al., 2009). L-α-aminobutyric acid (L-ABA, homoalanine) is a building block for the synthesis of important drugs such as ethambutol (antituberculosis drug), or levetiracetam and brivaracetam (antiepileptic drugs; Zhu et al., 2011). L-DOPA (levodopa) and several derivatives -including carbidopa- are among the most useful drugs for Parkinson’s disease treatment (Min et al., 2015; Gupta et al., 2019). L-5-hydroxytryptophan therapeutic effects include treatment of depression, chronic headache and insomnia (Hara and Kino, 2013). L- and D-biarylalanine-containing compounds have found different applications (e.g., dipeptidyl peptidase 4-, botulinum toxin- or amyloid-β-peptide aggregation- inhibitors; Ahmed et al., 2015). On the other hand, D-para-hydroxyphenylglycine (D-pHPG) and D-phenylglycine (D-PheGly) are utilized in the semi-synthesis of many different antibiotics (Martínez-Rodríguez et al., 2010c and references therein) (Table 2). Among these antibiotics, amoxicillin, cephalexin and ampicillin are included in the World Health Organization’s list of essential medicines. D-Phenylalanine is used in the preparation of nateglinide, a drug for the treatment of type 2 diabetes (Bettini et al., 2020). D-valine is used in the synthesis of tau-fluvalinate, a pyrethroid pesticide (Chen et al., 2016). D-amino acid mixtures have been shown to trigger biofilm disassembly in some bacterial species, promoting antibiotic sensitivity (Kolodkin-Gal et al., 2010; Dawe et al., 2017). As highlighted by the Nobel Prize Frances Arnold, important Protein Engineering efforts are also been paid to develop new ‘NcAA synthases’ for efficient, environmentally friendly production of valuable NcAAs (Almhjell et al., 2018). Nonetheless, many different enzymatic approaches have already been described in the past for NcAA production, which might give us some lessons from the past; these are interesting starting points for improvement, allowing new designs utilizing the immense enzymatic knowledge gained by the development of Green Chemistry.

**TABLE 1:** Some examples of free L-α-NcAAs or L-α-NcAA-containing compounds.

**Compound**	**Other names and utilization**	**References**
L-2-Aminobutyric acid	L-ABA, homoalanine. Key chiral intermediate for the synthesis of important drugs, such as levetiracetam or brivaracetam (antiepileptic drugs) and ethambutol (antituberculosis drug)	Zhu et al., 2011; Silva et al., 2019
L-Homophenylalanine	Precursor for the preparation of ACE and renin inhibitors(e.g., enalapril, lisinopril, quinapril, ramipril, trandolapril and benazepril)	Ahmad et al., 2009
L-Norvaline	Building block in the chemical synthesis of Perindopril, an antihypertensive drug (ACE inhibitor)	Park and Shin, 2015
L-Norleucine	Cost-effective residue-specific labeling of proteins	Anderhuber et al., 2016
L-*tert*-Leucine	Building block for HIV protease inhibitors and matrix metalloprotease inhibitors (MMPIs); organocatalysts	Xue et al., 2018; Agirre et al., 2019
Levodopa and derivatives	L-DOPA. In combination with carbidopa, used in Parkinson’s disease treatment since the 1960s. Etilevodopa, Melevodopa (more soluble L-DOPA prodrugs). Droxidopa (treatment of Parkinson/orthostatic hypotension)	Gupta et al., 2019
L-2-Chlorophenylglycine	Chiral synthon for the chemical synthesis of Clopidogrel, an antiplatelet agent used in the prevention and treatment of thrombosis	Saeed et al., 2017
L-Phosphinothricin	Glufosinate. Active ingredient of many commercial herbicides	Bartsch et al., 1996
L-Citruline	Pharmaconutrient	Eberhardt et al., 2014
L-Ornithine	Widely used to improve human health and reported to have beneficial effects on the liver and the heart	Wu et al., 2020
L-neo-Pentylglycine	Valuable synthons for organic synthesis	Gröger et al., 2006
L-Allysine ethylene acetal	Building block used for an production of Omapatrilat (antihypertensive drug)	Patel, 2001
*Trans*-4-Hydroxy-L-proline	Chiral synthon for the chemical synthesis for pharmaceuticals such as antiphlogistics, carbapenems and ACE- inhibitors	Shibasaki et al., 2000
L-3-Hydroxyadamantyl-glycine	Essential component of a type-2 diabetes drug (saxagliptin)	Patel, 2013
L-6-Hydroxynorleucine	A chiral intermediate required in the synthesis of omapatrilat (Vanlev), an antihypertensive drug	Patel, 2001
Ergothioneine	Antioxidant with therapeutic potential	Halliwell et al., 2018
L-Propargylglycine	Irreversible inhibitor of the enzyme cystathionine γ-lyase	Asimakopoulou et al., 2013; Weiser et al., 2015
β-(1-Azulenyl)-L-alanine	Spectroscopic probe for investigating protein dynamics and protein–protein interactions	Watkins et al., 2020
L-theanine	Taste-enhancing properties and probable health benefits, approved as GRAS ingredient by the FDA.	Mu et al., 2015, 2019; Yang et al., 2020
^18^F- and ^11^C -labeled NcAAs	Preclinical and clinical tumor PET/CT imaging	Sun et al., 2018
α-Vinylic AAs	L-Vinylglycine, useful for the study of PLP-dependent enzymes	Berkowitz et al., 2006
L-Cys derivatives	L-ethionine, *S*-phenyl-L-cysteine, potential applicability as an antiretroviral/protease inhibitor for HIV	Xu et al., 2019; Yu et al., 2019
L-pHFG and derivatives	Found in several peptidic natural products including the vancomycin group of antibiotics (e.g., vancomycin, chloroeremomycin, and complestatin) as well as other antimicrobial compounds such as ramoplanin	Al Toma et al., 2015
Fluorinated amino acids	Different applications in medicinal chemistry (e.g., amino acid decarboxylase inhibitors, bioisosteres, building block for different drugs, …)	Mei et al., 2020
L-Phe derivatives (*p*- ethynyl-, *p*- azido-, *p*-propargyloxy, *p*-*O*-pentynyl-)	Incorporation into proteins for electron paramagnetic resonance spectroscopy	Widder et al., 2019
*p*-and *m*-methoxy-L-Phe	Key intermediates for the synthesis of tamsulosin and HIV protease inhibitors, respectively.	Tork et al., 2019
*p*-Iodo-L-Phe	Site-specific incorporation into proteins for structure determination	Xie et al., 2004
*p*-Nitro-L-Phe	Building block for the synthesis of Melphalan, an anticancer drug	Ashnagar et al., 2007; Rosini et al., 2017
*p*-Fluoro-L-Phe	Building block for the synthesis of Melflufen (melphalan flufenamide), an anticancer drug	Cotton et al., 2019
*p*-Bromo-L-Phe	Intermediate in the production of several biarylalanines	Khorsand et al., 2017
*m*-(trifluoromethyl)-L-Phe	Integrated in kinesin KIFC1 inhibitors	Tork et al., 2019
Site-specifically incorporated reporter NcAAs	Allow examining local environments in peptides and proteins in solution (e.g., 4-cyano-L-phenylalanine, acridonylalanine, …)	Hostetler et al., 2018; Kearney et al., 2018
L-*p*-Boronophenylalanine	Boron neutron capture therapy (BNCT), a cancer therapy	Nakao et al., 1996
5-Different therapeutic effects (depression, chronic headache, and insomnia)	Hara and Kino, 2013
L–β–(thieno[3,2–b]pyrrolyl)–alanine	Substitution of Trp in proteins in the *E. coli* proteome for functional studies	Agostini et al., 2017
Substituted–L–arylalanines	Dipeptidyl peptidase 4 inhibitors, α4β7 integrin inhibitors, viral 3C–protease inhibitors and endothelin–converting enzyme inhibitors	Ahmed et al., 2015
Substituted L–pyridylalanines	Found in anticoagulants, dipeptidyl peptidase 1 inhibitors, leukocyte adhesion inhibitors, azaindoline anticancer agents, antidiabetics, or as structures for organocatalysts	Ahmed et al., 2016
L–Tryptophan analogs	Starting materials for chemical syntheses as well as probes for chemical biology. e.g., 4–Nitro–Trp, biosynthetic and chemical precursor to thaxtomin A, potentially useful agrochemical and a chemical precursor to the tumor–promoter indolactam VA; 4–cyano–tryptophan, fluorophore for imaging studies *in vitro* and *in vivo*; β–Alkyl Tryptophan Analogs, frequent components of useful natural products, biochemical probes, and therapeutics	Herger et al., 2016; Romney et al., 2017; Boville et al., 2018a, b; Dick et al., 2019
Metal–chelating NcAAs for metalloprotein engineering	e.g., (2,2-Almhjell and Mills, 2018

*Further NcAAs can be consulted in Blaskovich (2016), Zou et al. (2018), Narancic et al. (2019), and Hedges and Ryan (2020).*

**TABLE 2:** Some examples of D–α–AAs and D–α–AA–containing compounds.

**Compound**	**Other names and utilization**	**References**
D–Ala or D–Ser	Treatment of neuropsychiatric disorders	Tsai et al., 2006
D–Met	Preventing/reducing oral mucositis induced by radiation and chemotherapy for head and neck cancer	Hamstra et al., 2018
D–Phe	Modulator of L–phenylalanine–mediated amyloid formation, proposed as a therapeutic molecule in phenylketonuria. Used to obtain nateglinide for the treatment of type 2 diabetes; also used as analgesic and anti–stress agent	Singh et al., 2014; Bettini et al., 2020
D–Val	Precursor of different compounds. Fluvalinate (tau–fluvalinate) pyrethroid pesticide; it also forms part of D–penicillamine, actinomycin *D*, fungisporin and valinomycin (pharmaceutical drugs in clinical. Actinomycin *D* is employed clinically as chemotherapeutics for the treatment of highly malignant tumors	Chen et al., 2016
D–Lys	Reduction of renal uptake of radioactivity during scintigraphy and PRRT	Bernard et al., 1997
Different free D–α–AAs or their mixtures	Biofilm disassembly (e.g., D–leucine, D–methionine, D–tyrosine, and D–tryptophan at nanomolar concentration)	Kolodkin–Gal et al., 2010; Dawe et al., 2017
β–Chloro–D–Ala	Antibiotic, acts sinergically with D–cycloserine	David, 2001
Poly–D–Lys	Multi–compartment microfluidic device with covalently bound poly–D–Lysine greatly improved the differentiation and maturation of stem cell–derived neurons	Kamande et al., 2019
D–Cys	Generation of luminescent substrates for firefly luciferase (Luciferin and derivatives)	Godinat et al., 2014
D–Cyclohexylalanine	Chiral intermediate for the synthesis of thrombin inhibitor Inogatran, although lower effectivity than heparin	Patel, 2013
D–pHPG	Chiral intermediate for the synthesis of different antibiotics: Amoxicillin Cefadroxil, Cefatrizine, Cefprozil, Cefoperazone, Cefpiramide	Martínez–Rodríguez et al., 2010c
D–PG	Chiral intermediate for the synthesis of different antibiotics: Ampicillin, Cephalexin, Cefaclor, Pivampicillin, Piperacillin, Bacampicillin	Martínez–Rodríguez et al., 2010c
D–PG derivatives	e.g., 4–Fluoro–D–PG (chiral building block for potent h5–HT1D receptor agonist and HCV NS5B polymerase inhibitors), 4–Chloro–D–PG (key chiral synthon for a macrocyclic Hedgehog pathway inhibitor and a potent morpholinone MDM2 inhibitor)	Zhou et al., 2017
D–Ala	Building block of pharmaceutical drugs and synthesis of Alitame (artificial sweetener)	Kim and Shin, 2001; Han and Shin, 2018
D–Trp	Synthesis of Tadalafil (Cialis) for the treatment of male erectile dysfunction	Gouda, 2017
D–Ser/*O*–methyl–D–Ser	Building block of (R)–lacosamide (Vimpat), an antiepileptic drug	Aratikatla and Bhattacharya, 2020
D–*tert*–Leu	Synthesis of antitumor, anti–inflammatory, and antiviral agents.	Cheng et al., 2018
D–2–ABA	Synthesis of antibiotics, angiotensin–converting enzyme 2 inhibitors, brain–permeable polo–like kinase–2 (Plk–2) inhibitors, matrix metalloproteinase inhibitors and antiproliferatives	Chen et al., 2017
D–Fluoroalanine	Inhibition of bacterial alanine racemase	Mei et al., 2020
D–Ala/D–Leu	Neuroprotective and neuroregenerative potential	Liska et al., 2018
D–Trp–containing peptides (with additional D–α–AAs)	Immunosuppressors (e.g., Thymodepressin^®^) and other hemosuppressive Thymodepressin^®^ analogs	Deigin et al., 2020
D-Homophenylalanine	Building block for the synthesis of highly potent factor XA inhibitors	Stürzebecher et al., 2007
D-Phe derivatives (D-arylalanines)	Building blocks in the synthesis of many pharmaceuticals, including antibiotics, antidiabetics and chemotherapeutic agents	Walton et al., 2017; Zhu et al., 2019
D-(2,4,5-trifluoro)-Phe	Key precursor of the antidiabetic sitagliptin	Parmeggiani et al., 2019a
D-(5,5,5-trifluoro)-Norvaline	Intermediate of avagacestat (BMS-708163), a potent inhibitor of γ-secretase	Hanson et al., 2013
D-*m*-(trifluoromethyl)-Phe	Key chiral intermediate for (*R*)-PFI-2, a potent inhibitor for SETD 7, involved in multiple cancer-cancer related	Tork et al., 2019
D-*p*-methyl-Phe	Incorporated into Pin1 inhibitors, anti-inflammatory formyl peptide receptor 1 antagonist	Tork et al., 2019
Substituted-D-arylalanines	Synthesis of biarylalanines through chemoenzymatic reaction (botulinum toxin inhibitors, amyloid-β-peptide aggregation inhibitors, kinesin-14 motor protein KIFC1 inhibitors or and reverse cholesterol transport facilitators	Ahmed et al., 2015
Substituted D-Trp derivatives	Building blocks for mitragynine, or inhibitors of breast cancer resistance protein or necrostatins	Parmeggiani et al., 2019b
(*R*)-2-amino-3-(7-methyl-1*H*-indazol-5-yl)-propanoic acid	Key intermediate for the synthesis of antagonists of calcitonin gene-related peptide receptors, potentially useful for migraine and other maladies	Patel, 2018

*Further information can be consulted in Martínez-Rodríguez et al. (2010c); Grishin et al. (2020), and Pollegioni et al. (2020).*

## Multienzymatic Cascades for the Production of NcAAs

NcAAs chemical synthesis continues receiving at present huge attention in the literature due to the relevance of these compounds (Xue et al., 2018; Mei et al., 2020; Zou et al., 2020). On the other hand, the search for sustainable processes to decrease the environmental impact of industrial processes (Wenda et al., 2011; Sheldon and Brady, 2018) is probably the main reason boosting the development of multienzymatic cascade (MEC) reactions. The unprecedented development in bioinformatics, metagenomics and *de novo* design coupled with protein engineering (i.e., directed evolution and high-throughput screening) during the last decade have resulted in a massive diversification on the enzymes available for synthetic Chemistry and Biology (Devine et al., 2018). These advances have accelerated the arrival of the “Fourth Wave of Biocatalysis” (Bornscheuer, 2018) or the so-called “Golden Age of Biocatalysis” (Devine et al., 2018); combinations of enzymes -whether in cascade reactions or via metabolic engineering- brings together many beneficial features which might convert this strategy as the ‘first choice’ to advance in the biotransformation field (Bornscheuer, 2018). The huge research efforts in this field are reflected by different reviews from the last decade on general operational and functional aspects on different MECs, some of them also including disseminated information on AA production using chemoenzymatic and multienzymatic systems (Lopez-Gallego and Schmidt-Dannert, 2010; Hall and Bommarius, 2011; Ricca et al., 2011; Oroz-Guinea and García-Junceda, 2013; France et al., 2017; Quin et al., 2017; Devine et al., 2018; Schrittwieser et al., 2018; Sperl and Sieber, 2018; Wu and Li, 2018; Cutlan et al., 2019; Hwang and Lee, 2019; Giannakopoulou et al., 2020). The interest on MECs in biotransformation and biomedical engineering is thus clear, being an attractive alternative for the production of biofuels, pharmaceuticals and fine chemicals (Hwang and Lee, 2019). A short summary of different MECs developed for the production of NcAAs during the last decades can be consulted in Table 3.

**TABLE 3:** Summary of different MECs for the production of NcAAs described in the literature.

**Multienzymatic cascade**	**MEC example to L-NcAA**	**MEC example to D-NcAA**
Hydantoinase Process	DHYD + HR + NSAR + LCAR	DHYD + HR + DCAR
Amidohydrolase Process	L-NxAH + NSAR	D-NxAH + NSAR
Amidase Process	L-AMID + ACLR	D-AMID + ACLR
Amino acid oxidase-based MECs	DAAO/catalase + L-TA + TPL	TrpS + LAAO
Amino acid dehydrogenase-based MECs	DAAO/catalase + LAADH + CRS	LAAO/catalase + DAADH + CRS
Ammonia lyase-based MECs	PAL + DAOO	PAL + LAAD
Transaminase-based MECs	D-TA + L-PheDH + CRS	LAAD + D-TA
Lipase-containing MECs	Lipase + protease	—
Tyrosine phenol lyase-containing MECs	TD + TGDH + TPL	—
Tryptophan synthase-containing MECs	D-threonine aldolase + TrpS + AR	—
Amino acid ester racemase/esterase system	ACLR-homolog with AAER activity + esterase	—

*An illustrative example is shown, but there are other possible combinations. It is advisable that the reader consults the specific example in the corresponding section to determine whether (i) isolated, enantiopure and/or enantioenriched AAs are obtained, and (ii) the MEC is a general method suitable for the production of different NcAAs or specific NcAAs. Additional steps might also be necessary, such as separation of other compounds produced in the reactions, or chemical steps to convert the systems into chemoenzymatic DKR methods. DHYD, D-hydantoinase; HR, hydantoin racemase; NSAR, *N*-succinyl-amino acid racemase; DCAR, D-carbamoylase; LCAR, L-carbamoylase; L-NxAH, L-*N*-Substituted amidohydrolase (e.g., L-acylase, LCAR or L-succinylase); D-NxAH, D-*N*-Substituted amidohydrolase (e.g., D-acylase, DCAR, or D-succinylase); L-AMID, L-amidase (e.g., L-proline amidase or L-amidase); ACLR, α-amino ϵ-caprolactam racemase; D-AMID, D-amidase (e.g., D-aminopeptidase); DAAO, D-amino acid oxidase; L-TA, L-enantioselective transaminase; LAADH, L-amino acid dehydrogenase (e.g., LeuDH, GluDH); CRS, cofactor recycling system; LAAO, L-amino acid oxidase; TrpS, tryptophan synthase; DAADH, D-amino acid dehydrogenase; PAL, phenylalanine ammonia lyase; LAAD, L-amino acid deaminase; D-TA, D-enantioselective transaminase; L-PheDH, L-phenylalanine dehydrogenase; TD, toluene dioxygenase; TGDH, toluene *cis*-glycerol de-hydrogenase; TPL, tyrosine phenol lyase; AR, alanine racemase; AAER, amino acid ester racemase.*

The plausible combinations of MEC are huge and their operational aspects have been reviewed in detail in the last lustrum. Firstly, MEC can be developed using purified proteins, whole cells or cell-free extracts. The spatial organization of MECs have also received special attention, since it affects important operational parameters such as substrate/product diffusion, the availability of cofactors or the probable interference or inhibition of cofactors among the different enzymes used in the reactions (Quin et al., 2017; Bugada et al., 2018). In this sense, many diverse multienzyme arrangements have been proposed, such as fusion proteins (e.g., including a peptide linker), nucleic acid-based or protein-based scaffolds, co-immobilization, Vesicle-based or protein-based encapsulation, or even repurposed cellular organelles (Bugada et al., 2018; Hwang and Lee, 2019). Also, different recent reviews have described different approaches and materials suitable for MEC immobilization (Zdarta et al., 2018; Hwang and Lee, 2019; Ren et al., 2019; Romero-Fernández and Paradisi, 2019). Since many MECs rely on the regeneration of cofactors for continuous operation, its selection is also important; several regeneration systems have been studied from the middle of 20th century, and different alternatives are available, such as formate dehydrogenase (FDH), glucose dehydrogenase (GDH) or NADH Oxidase (NOX) among many others (De Wildeman et al., 2007; Hall and Bommarius, 2011; Tassano and Hall, 2019). This complex scenario makes difficult to efficiently categorize MECs, but the reader is referred to the seminal work by Kroutil’s group to envision different parameters which can be used for classification of biocatalytic artificial cascades (e.g., number of steps/catalysts, chronology, topology, types of catalysts used; Schrittwieser et al., 2018).

### Hydantoinase Process

**FIGURE 1:**
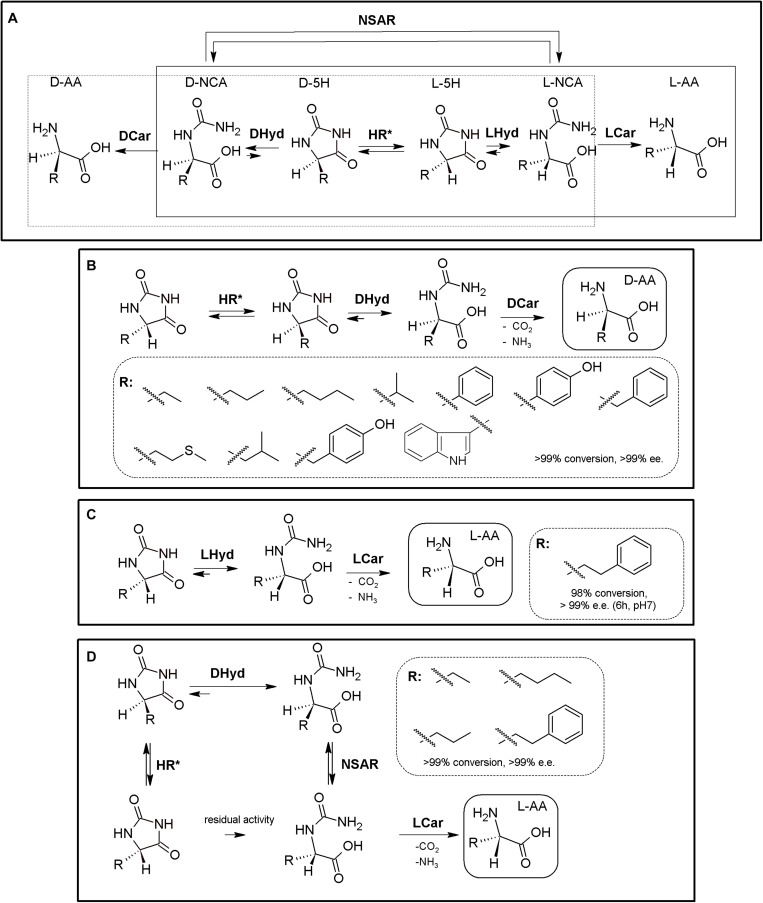
The Hydantoinase Process. **(A)** General scheme on the different possibilities for the Hydantoinase Process. The “D-system” appears contained in the dashed line, whereas the “L-system” is remarked by a full line. Inclusion of an NSAR enzyme highly improves the “L-system.” HR*: hydantoin racemase (or chemical racemization when favored); DHyd, D-activity of hydantoinase; DCar, D-carbamoylase; LHyd, L-activity of hydantoinase; LCar, L-carbamoylase; NSAR, *N*-carbamoyl-racemase promiscuous activity of *N*-succinyl-racemase (Martínez-Rodríguez et al., 2010b, 2020). D-AA, D-α-amino acid. D-NCA, *N*-D-carbamoyl-α-amino acid. D-5H, D-5-monosubstituted hydantoin. L-AA, L-α-amino acid. L-NCA, *N*-L-carbamoyl-α-amino acid. L-5H, L-5-monosubstituted hydantoin. **(B)** Conversion of different racemic 5-monsubstituted hydantoins till the corresponding enantiopure D-α-AA using the Hydantoinase Process (D-system). Recombinant *E. coli* cells containing two different recombinant polycistronic systems using *Agrobacterium* enzymes were used. HR*: hydantoin racemase (or chemical racemization when favored); DHyd, D-activity of hydantoinase; DCar, D-carbamoylase (Martínez-Gómez et al., 2007). **(C)** Bioconversion of L-homophenylanine hydantoin derivative using recombinant *E. coli* cells. Coexpression of hydantoinase from *Brevibacillus agri* (Hyd) and L-carbamoylase (LCar) from *B. kaustophilus* was carried out. Hyd from *Brevibacillus agri* is highly D-enantioselective, but possess residual L-activity for this specific substrate (Kao et al., 2008). **(D)** “Double-racemase Hydantoinase Process” for the production of enantiopure L-α-AAs. Different combinations of purified and immobilized D-hydantoinase (DHyd) and hydantoin racemase (HR) from *Agrobacterium* species, *N*-succinyl-amino acid racemase (NSAR) from *Geobacillus kaustophilus* and L-carbamoylase (LCar) from *Geobacillus stearothermopillus* allowed the production of different enantiopure NcAAs; L-Met and L-Val were also efficiently produced with this system (Rodríguez-Alonso et al., 2015, 2016, 2017).

The “Hydantoinase Process” is a cheap and environment-friendly enzymatic cascade for the potential production of virtually any enantiopure α-AA. This process is known for more than four decades, and received its name from the ability of D-hydantoinases (dihydropyrimidinase, E.C. 3.5.2.2) to hydrolyze a wide spectrum of D,L-5-monosubstituted hydantoins. The latter compound was converted till the corresponding *N*-carbamoyl-D-α-AA, which could be afterward hydrolyzed chemically to the corresponding D-α-AA. D-hydantoinase was afterward coupled with a stereospecific D-carbamoylase (E.C. 3.5.1.77) to obtain the corresponding D-α-AA starting from a racemic mixture of 5-monosubstituted hydantoins (Figure 1A), taking advantage of the spontaneous racemization of these substrates under certain conditions (see below; Grifantini et al., 1998; Slomka et al., 2017). Its principal application was the production of D-pHPG and D-PheGly (precursors of Ampicillin and Amoxicillin, Table 2), but it has been applied industrially for the production of different enantiopure AAs by DSM, Evonik, Kanegafuchi or Recordati (Bommarius et al., 1998; Wilms et al., 2001; Wenda et al., 2011). Despite its relevance, DKRs accomplished through this process were initially limited to 5-monosubstituted hydantoins for which a fast spontaneous racemization was favored since racemization of most hydantoins is usually a very slow process; chemical racemization is highly dependent on the pH, temperature and other factors (such as bulkiness) of the substituent in the 5-position of these precursors (Pietzsch and Syldatk, 2002). This enzymatic tandem was enhanced to increase its substrate scope, allowing the production of additional D-α-AAs by inclusion of a third enzyme together the original hydantoinase/carbamoylase, namely hydantoin racemase (E.C. 5.1.99.5, Martínez-Rodríguez et al., 2004). This third enzyme allowed extending the use of the hydantoinase/carbamoylase tandem to 5-monosubstituted hydantoin substrates for which chemical racemization is not favored (Wilms et al., 2001; Martínez-Rodríguez et al., 2002; Martínez-Gómez et al., 2007). Total conversion and 100% enantiopure D- or L-α-AAs can thus be obtained when a HR racemases the remaining non-hydrolyzed 5-monosubstituted hydantoin (Figure 1A).

As most hydantoinases exhibit clear D-enantioselectivity (Martínez-Rodríguez et al., 2010a), this MEC has been mainly applied for the production of enantiopure D-α-AAs (Figure 1A, dashed line). Different D-α-AAs were produced using whole cells of recombinant polycistronic systems containing D-hydantoinase, D-carbamoylase and hydantoin racemases from *Agrobacterium* species (Figure 1B). 0.3 M D,L-5-(2-methylthioethyl)hydantoin (52.3 g⋅L^–1^) was totally converted after 6 h till D-Met using this approach (0.25 g of cells⋅mL^–1^, pH 8; Martínez-Gómez et al., 2007). On the other hand, hydantoinases usually present a “residual” L-activity that can be coupled to an L-stereospecific carbamoylase (Figure 1A, full line) (E.C.3.1.5.87) (Wilms et al., 2001; May et al., 2002; Kao et al., 2008); protein engineering allowed obtaining a preferential L-hydantoinase activity toward L-5-methyl-thio-ethyl hydantoin, highly enhancing the production of L-methionine (May et al., 2000). Recombinant *E. coli* cells coexpressing thermostable hydantoinase (dihydropyrimidinase) from *Brevibacillus agri* and L-carbamoylase from *Bacillus kaustophilus* allowed *c*onversion yields of 98% starting from enantiopure L-substrate at pH 7.0 (Figure 1C, Kao et al., 2008). Using racemic substrate, 43% conversion was achieved, since the D-carbamoyl-derivative accumulates in the reaction and the chemical racemization of this substrate is not favored. On the other hand, the system could be reused at least 8 times without noticeable loss of activity (Kao et al., 2008).

This D-hydantoinase/L-carbamoylase system was further expanded by coupling with an *N*-succinyl-amino acid racemase (NSAR, E.C.3.1.5.87), since the latter enzyme allows the racemization of the D-*N*-carbamoyl-α-AA produced by hydantoinase. Thus, *in situ* conversion producing L-*N*-carbamoyl-α-AA in the reaction medium occurs, which can be further hydrolyzed by L-carbamoylase to the corresponding L-α-AA (Figure 1D). However, this system still depends on the L-residual enantioselective activity of D-hydantoinase for those substrates whose chemical racemization is not favored. Different reports on the hydantoinase/NSAR/L-carbamoylase system have been reported (Bommarius et al., 2002; Lo et al., 2009). Engineered dihydropyrimidinase from *Brevibacillus agri* (L159V mutant), *Bacillus kaustophilus* L-carbamoylase and *Deinococcus radiodurans* NSAR were purified, and allowed the production of L-homophenylalanine (90% yield, 5 h, Lo et al., 2009). This L-enantiospecific MEC system has further been expanded by inclusion of a hydantoin racemase, speeding up the process by racemization of the remaining L-5-monosubstituted hydantoin for substrates whose chemical racemization is not favored (Rodríguez-Alonso et al., 2015, 2016, 2017; Figure 1D). Using purified and immobilized enzymes, the proposed “double-racemase Hydantoinase Process” was efficiently applied for the synthesis of different NcAAs (L-norvaline, L-norleucine, L-ABA, L-homophenylalanine). The immobilized system could be reused 15 times, retaining 80% of the initial activity (Rodríguez-Alonso et al., 2017).

Thus, enantiopure D-or L-α-AAs can be obtained by different combinations of hydantoinases (Altenbuchner et al., 2001), carbamoylases (Martínez-Rodríguez et al., 2010b), NSARs (Martínez-Rodríguez et al., 2020), and hydantoin racemases (Martínez-Rodríguez et al., 2004). This process has also been expanded for the production of enantio-enriched β-AAs (Martínez-Gómez et al., 2012; Rudat et al., 2012).

### Amidohydrolase Process

**FIGURE 2:**
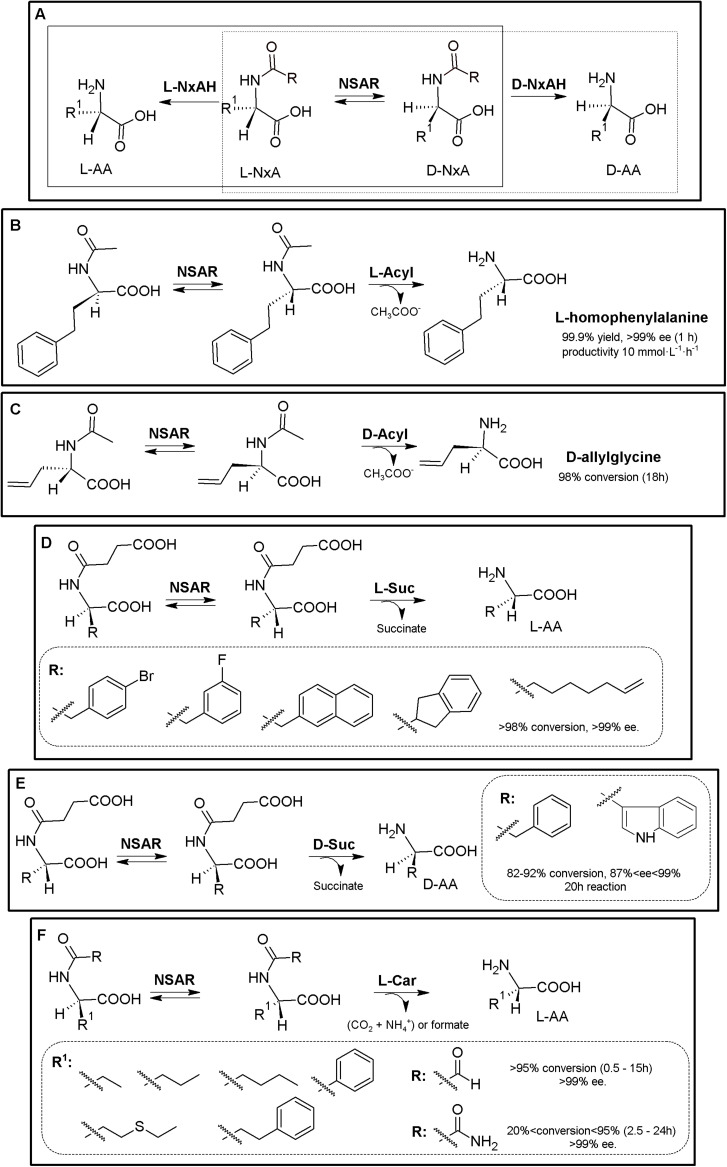
Amidohydrolase process. **(A)** General scheme for the “Amidohydrolase Process” (D-system, dashed line; L-system, full line). L-NxAH, *N*-substituted L-stereospecific amidohydrolase (e.g., L-acylase, L-carbamoylase, L-succinylase). D-NxAH, *N*-substituted D-stereospecific or stereoselective amidohydrolase ((e.g., D-acylase, D-carbamoylase, D-succinylase). R: Acyl, NSAR/Acylase tandem (original acylase process). R: carbamoyl, NSAR/carbamoylase tandem. R: formyl, NAAR/carbamoylase tandem. R: succinyl, NSAR/succinylase tandem (Martínez-Rodríguez et al., 2020). **(B)** L-homophenylalanine production by NSAR/L-acylase tandem. Whole cell biocatalyst containing *N*-succinyl-amino acid racemase (NSAR) and L-acylase (L-Acyl) from *Deinococcus radiodurans* were used, starting from racemic *N*-acetyl-L-homophenylalanine (Hsu et al., 2007). **(C)** D-allylglycine production by NSAR/D-acylase tandem, consisting of pure D-acylase (D-Acyl, Chirotech Technology Ltd.) and an engineered *N*-succinyl-amino acid racemase from *Amycolatopsis* sp (NSAR, G291D/F323Y mutant; Baxter et al., 2012). **(D)** Production of enantiopure L-α-AA using an NSAR/L-succinylase system, using enzymes from *Geobacillus kaustophilus* overexpressed in a whole cell system (Masutoshi et al., 2016). **(E)** Production of enantio-enriched D-Phe and D-Trp using a NSAR/D-succinylase system. The enzymes were cloned from *Cupriavidus* sp. and *Geobacillus stearothermophilus*, respectively (Sumida et al., 2018). **(F)** Production of enantiopure L-α-AA using purified NSAR/L-carbamoylase system. Purified NSAR from *Geobacillus kaustophilus* and L-carbamoylase from *Geobacillus stearothermophilus* were applied for the DKR of different racemic *N*-carbamoyl- and *N*-formyl-α-AAs. R^1^, lateral chain of the corresponding AA. R, *N*-substituent (Soriano-Maldonado et al., 2014a, b).)

The original Industrially used “Acylase Process” (Degussa, now Evonik) consisted of a KR of *N*-acetyl-α-AA using L- (E.C. 3.5.1.4) or D-stereospecific acylases (E.C. 3.5.1.81)^2^. Coupling of an NSAR with an stereospecific acylase produces a bienzymatic DKR system allowing the production of different enantiopure L- or D-α-AAs starting from inexpensive *N*-acetyl-α-AAs (Figure 2A; May et al., 2002; Hsu et al., 2006; Baxter et al., 2012). Based on this process, and taking advantage of (i) the substrate promiscuity of NSAR enzymes toward different *N*-substituted-α-AAs (NxAs), (ii) the existence of different enantioselective or stereospecific amidohydrolases (NxAH) under E.C. 3.5.1 enzyme group, and (iii) the substrate promiscuity shown by these enzymes (e.g., D- and L-carbamoylases, Martínez-Rodríguez et al., 2010b), coupling of an NSAR with a stereospecific NxAH allows the production of D- or L-α-AAs starting from many different substrates (e.g., *N*-succinyl-, *N*-acetyl-, *N*-carbamoyl-, *N*-chloroacetyl-, *N*-butyryl-, *N*-propyl-, *N*-benzoyl, or *N*-formyl-AAs; Martínez-Rodríguez et al., 2020 and references therein). Thus, the “Amidohydrolase Process” is a more general nomenclature which encompasses the use of a promiscuous and stereospecific D- or L-amidohydrolase together an NSAR enzyme, generating different NSAR/NxAH tandems. Different DKR MECs for production of enantiopure D- or L-α-AAs arise from these combinations, allowing the use of different NxAs, as a result of the broad substrate promiscuity of NSARs and NxAHs (Figure 2A). So far, different NSAR/Acylase, NSAR/carbamoylase or NSAR/succinylase tandems have been used or proposed (Martínez-Rodríguez et al., 2020 and references therein; Figures 2B–F). Metal requirement/compatibility of these MECs needs to be studied in order to assess optimal conditions of the different MECs. Furthermore, since all these enzymes have shown broad substrate promiscuity, proper selection of the substrate of NSAR/NxAH tandems can enhance conversion rates (Soriano-Maldonado et al., 2014a, b).

An *E. coli* whole cell system comprising a NSAR/L-acylase from *Deinococcus radiodurans* was used to produce L-homophenylalanine with a 99.9% yield and over 99% e.e. (in 1 h), with a productivity of 10 mmol⋅L^–1^⋅h^–1^ (Figure 2B, Hsu et al., 2007). Engineered NSAR (G291D/F323Y mutant) from *Amycolatopsis* sp., together with D-acylase (Chirotech Technology Ltd) allowed conversion of *N*-acetyl-D,L-allylglycine (50 g⋅L^–1^) into D-allylglycine in 18 h with a 98% conversion (Figure 2C, Baxter et al., 2012). Whole cell systems containing NSAR and L-succinylase from *Geobacillus kaustophilus* were successfully applied for the synthesis of L-4-bromophenylalanine, L-3-fluorophenylalanine, L-2-naphthylalanine, L-2-indanylglycine, and L-6-heptenylglycine (conversion over 98%, >99.9% e.e.) (Figure 2D, Masutoshi et al., 2016). An NSAR/D-succinylase system has also been reported (Sumida et al., 2016, 2018). However, the enantiomeric excess of D-Trp and D-Phe produced by the biocatalysts was lower than 95%; the authors suggested that D-succinylase from *Cupriavidus* sp. is enantioselective and also recognize the L-isomer, concluding that this system needs still development for improving the enantioselective character of D-succinylase (Figure 2E). Purified and immobilized NSAR from *Geobacillus kaustophilus* and L-carbamoylase from *Geobacillus stearothermophilus* were applied for the DKR of different racemic *N*-carbamoyl- and *N*-formyl-α-AAs (Figure 2F; Soriano-Maldonado et al., 2014a, b). Unexpectedly, *N*-formyl-substrates were recognized more efficiently than *N*-carbamoyl-α-AAs. Total conversion till 15 mM of L-ABA, L-norleucine, L-norvaline, or L-homophenylalanine was achieved in less than 2 h (Soriano-Maldonado et al., 2014a). A preparative scale reaction was also conducted; 0.5 M of racemic *N*-formyl-aminobutyric acid could be converted in 85 h (at low enzyme concentrations; 2.0 μM L-carbamoylase and 12.0 μM NSAR, CoCl_2_ 0.25 mM). A productivity of 16 mmol L-norleucine L^–1^⋅h^–1^ (yield > 99%; e.e. 99.5%), with no inhibition at high substrate or product concentrations using immobilized NSAR/L-carbamoylase (Soriano-Maldonado et al., 2014b).

The Amidohydrolase Process is an example of the expansion possibilities of well-stablished MECs (Martínez-Rodríguez et al., 2020). As way of example, oxyfunctionalized AAs [L-methionine-(*S*)-sulfoxide and different γ-hydroxy-AAs] were obtained by using a NSAR/L-acylase tandem coupled with the stereoselective isoleucine dioxygenase from *Bacillus thuringiensis* (Supplementary Figure S1, Enoki et al., 2016). L-methionine-(*S*)-sulfoxide with 97% yield and 95% d.e. was produced starting from racemic *N*-acetyl-Met. Hydroxylation of AAs is one of the numerous theoretical MEC expansion possibilities for the production of oxo-functionalized AAs (Hibi et al., 2012; Smirnov et al., 2012; Busto et al., 2014; Peters and Buller, 2019).

### Amidase Process

**FIGURE 3:**
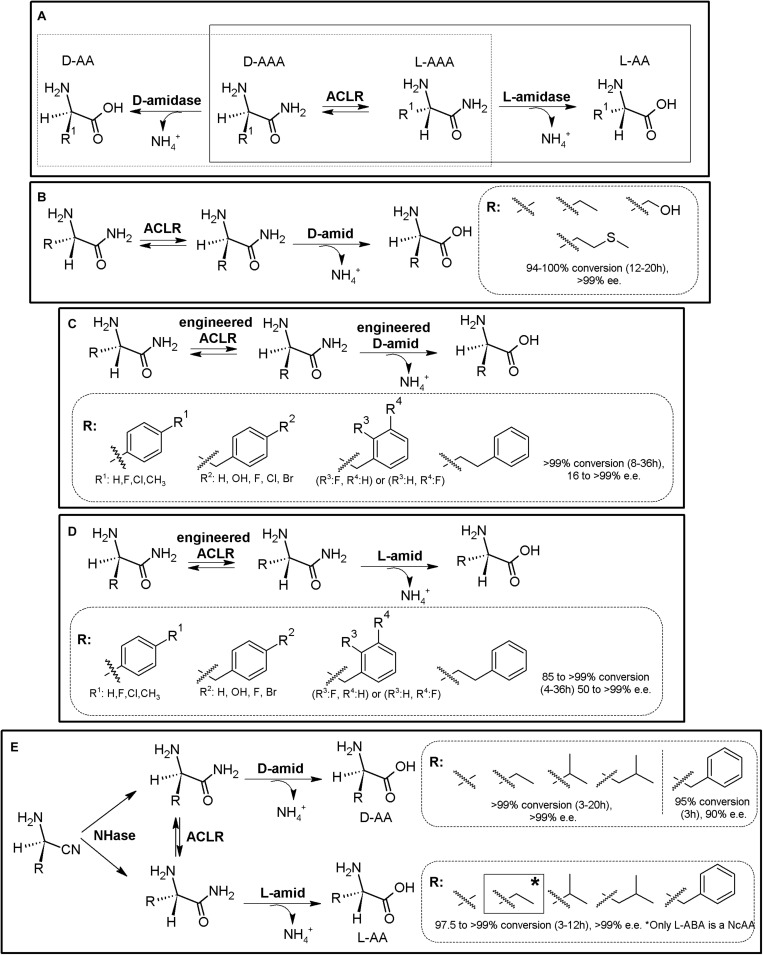
Amidase process. **(A)** General scheme on the Amidase Process (D-system, dashed line; L-system, full line). D-amidase (e.g., D-aminopeptidase, D-amino acid amidase, alkaline D-peptidase or R-amidase). ACLR, PLP-dependent α-amino ϵ-caprolactam racemase. L-amidase (e.g., L-amino acid amide hydrolases such as LaaA and LaaA_Bd._ *A chemoenzymatic approach is also possible in a two-step process (Yamaguchi et al., 2007). **(B)** Synthesis of different enantiopure D-α-AAs from the corresponding L-α-AA amides using the Amidase Process (D-system), using purified D-aminopeptidase from *Ochrobactrum anthropi* (D-amid) and ACLR from *Achromobacter obae* (Yamaguchi et al., 2007). Racemic AA-amides can also be used with this system. **(C)** Synthesis of different enantio-enriched and enantiopure aromatic D-α-AAs, using an engineered thermostable D-aminopeptidase from *Ochrobactrum anthropi* and engineered ACLR from *Achromobacter obae* (Yasukawa and Asano, 2012). **(D)** Production of different enantio-enriched and enantiopure aromatic L-α-AAs, using engineered ACLR from *Achromobacter obae* and L-amino acid amidase from *Brevundimonas diminuta* in *E. coli* (Yasukawa and Asano, 2012). **(E)** Expansion of the Amidase Process by inclusion of a nitrilase. Enantioselective nitrilase (NHase), coupled to ACLR from *Achromobacter obae*, D-aminopeptidase from *Ochrobactrum anthropi* or L-amino acid amidase from *Brevundimonas diminuta* allowed the production of D- or L-α-AA (Yasukawa et al., 2011; Yasukawa and Asano, 2012).

As for the Amidohydrolase Process, the “Amidase Process” was initially conceived as a KR process taking advantage of stereoselective D-aminopeptidases (EC 3.4.11.19) or the enantioselective L-amidase activity of different enzymes (such as L-proline amidase or L-amidase from the formamidase family (Sonke et al., 2005). Racemic α-AA-amide substrate can thus be deracemized into enantiopure or enantioenriched D- (or L-α-AAs) and the corresponding non-hydrolyzed amide, using D- (or L-) stereospecific “amidases” (Figure 3A). Since the non-hydrolyzed amide can be chemically racemized, 100% conversion can be obtained using a chemoenzymatic approach. PLP-dependent α-amino ϵ-caprolactam racemase (ACLR; E.C. 5.1.1.15) naturally catalyzes the racemization of α-amino-ε-*caprolactam* (ϵ-ACL), and was long ago applied for the production of L-lysine coupled to an L-lysine-lactamase (Fukumura, 1977). However, ACLR from different organisms have been proved to be active toward different NcAA amides (Asano and Yamaguchi, 2005a). Thus, coupling of an ACLR with D- or L-specific amidases allows for the production of enantiopure AAs by enzymatic DKR (Figure 3A).

Coupling of D-aminopeptidase from *Ochrobactrum anthropi* and ACLR from *Achromobacter obae* allowed the production of D-Ala, D-ABA, D-Ser, and D-Met (Figure 3B, Asano and Yamaguchi, 2005b; Yamaguchi et al., 2007); As way of example, 45 mM L-alanine amide was converted to D-alanine (7 h, yield > 99.7%; Asano and Yamaguchi, 2005b). Coupling of purified ACLR from *Achromobacter obae* mutant (L19V/L78T) obtained by directed evolution, together with a thermostable mutant of D-amino acid amidase from *Ochrobactrum anthropi* SV3 allowed for the biosynthesis of different enantio-enriched D-PheGly and D-Phe derivatives (Figure 3C, Yasukawa and Asano, 2012). On the other hand, the optical purity of this system varied greatly for aromatic substrates, with e.e. values ranging 16–99%. These results differed to the results presented previously on aliphatic substrates (Figure 3C, Yamaguchi et al., 2007), suggesting enantioselectivity of D-amino acid amidase, as it was observed with D-succinylase (Figure 2E). Production of enantiopure L-Ala, L-Leu, and L-Met (100% yield, >99% e.e.) was reported by coupling ACLR from *Achromobacter obae* with L-Amino acid amide hydrolase from *Pseudomonas azotoformans* (Yamaguchi et al., 2007). This strategy was afterward proved useful for the production of different L-α-AAs. By overexpression of mutated ACLR together with L-amino acid amidase from *Brevundimonas diminuta* in *E. coli*, efficient production of various enantio-enriched (*S*)-phenylalanine derivatives was achieved (Figure 3D, 99% yield, 90–98% e.e.). This system allowed production of L-homophenylalanine, which continuously precipitated in the reaction mixture (>99% yield, 98% e.e., 12 h; Yasukawa and Asano, 2012). On the other hand, lower e.e. values were also obtained for other aromatic compounds.

The Amidase Process has been further expanded by including non-stereoselective nitrile hydratase (NHase, EC 4.2.1.84) for the production of different highly enantio-enriched and enantiopure D- and L-α-AAs (Figure 3E; Yasukawa et al., 2011; Yasukawa and Asano, 2012). A MEC using purified nitrile hydratase from *Rhodococcus opacus* (RoNHAse), D-aminopeptidase from *Ochrobactrum anthropi* and ACLR from *Achromobacter obae* allowed total conversion of racemic α-aminobutyronitrile in 6 h and 30°C to D-ABA (e.e., >99%) (Figure 16, Yasukawa et al., 2011). D-Phe was also produced afterward using the same strategy (Yasukawa and Asano, 2012). L-ABA was synthesized by combination of RoNHAse, ACLR from *Achromobacter obae* and L-amino acid amidase from *Brevundimonas diminuta* (e.e., >99%) (Yasukawa et al., 2011). Other different D- and L-α-AAs were produced with these systems (> 99% Yield, e.e. 97 to >99%; Figure 3E, Yasukawa et al., 2011). Remarkably, RoNHase and ACLR were reported to suffer inhibition by the substrate α-amino nitrile, and thus, substrate concentrations need to be taken into account if using this MEC combination (Yasukawa and Asano, 2012). A recent study has showed that the ACLR from *Ochrobactrum anthropi* also racemizes α-AA esters (Frese et al., 2018). This activity opens up new enzyme combinations for the synthesis of enantiopure α-AAs, coupling ACLR with stereospecific esterases (see section “AAER/Esterase System”).

### Amino Acid Oxidase-Based MECs

**FIGURE 4:**
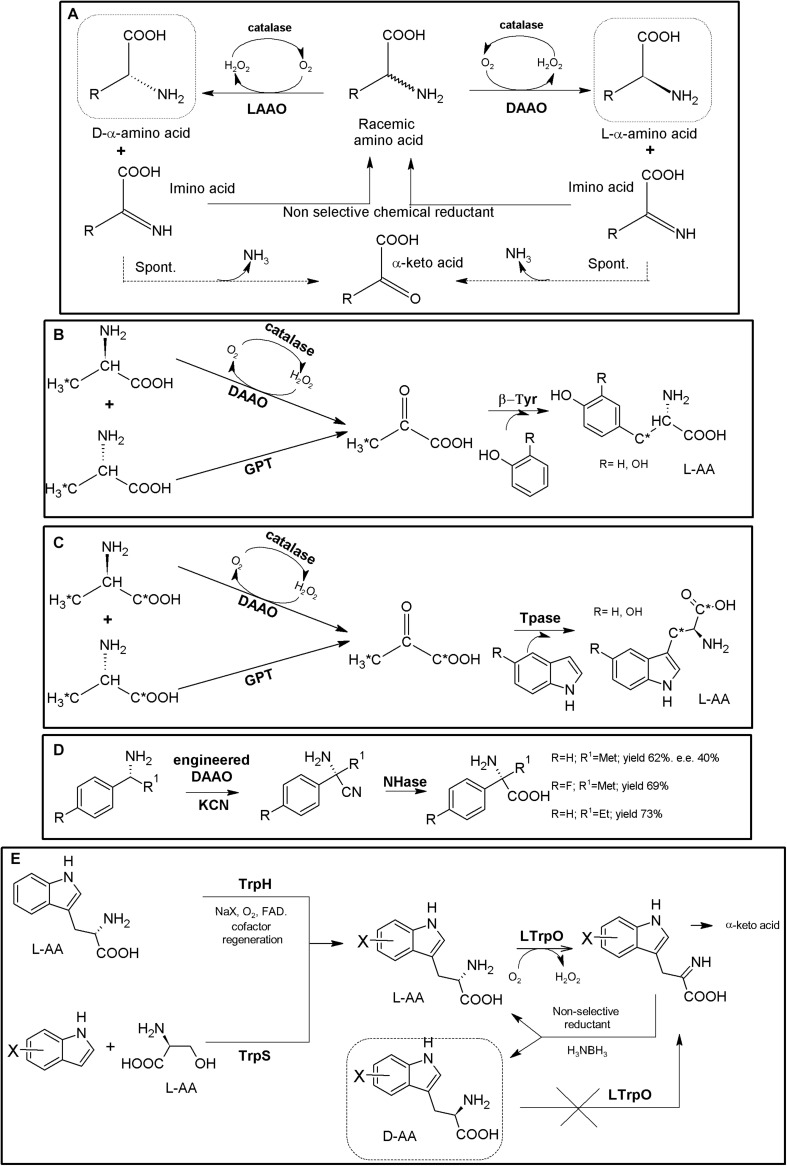
AAO-based multienzymatic cascades. **(A)** General scheme for enzymatic KR and chemoenzymatic DKR of AA racemates starting from AAO/catalase systems. **(B)** Enzymatic synthesis of ^11^C L-Tyr (R = H) and L-DOPA (R = OH). A MEC consisting of DAAO from porcine kidney, Glutamic-pyruvic transaminase (GPT) (from porcine heart, catalase from bovine liver and β-tyrosinase (β-Tyr, tyrosine phenol lyase) purified from *Citrobacter intermedius* were successfully applied. The radio-labeled atom is highlighted by an asterisk (Bjurling et al., 1990). **(C)** Enzymatic synthesis of 1-^14^C- or 3-^14^C-tryptophan (R = H) or 1-^14^C- or 3-^14^C 5-hydroxytryptophan (R = OH). It was conducted with a MEC consisting of DAAO, Glutamic-pyruvic transaminase (GPT), catalase and tryptophanase (Tpase, L-tryptophan indole-lyase). An asterisk highlights the relative positions of the radio-labeled atoms (Pająk et al., 2018). **(D)** Synthesis of NcAAs starting from primary amines; a MEC comprising mutant porcine kidney DAOO and nitrilase AY487533 (NHase) was used (Kawahara et al., 2017). **(E)** Two-step chemoenzymatic syntheses of halogenated D-Trp derivatives. Combination of biocatalytic halogenation by tryptophan halogenase (TrpH) or Trp synthase (TrpS) with L-Trp oxidase (LTrpO), followed by non-selective reduction allowed to obtain D-Trp derivatives (Schnepel et al., 2019).)

Amino acid oxidases (AAOs) are important biotechnological flavoenzymes catalyzing the oxygen-dependent oxidative deamination of D- or L-α-AAs, resulting in α-keto acids, ammonia and hydrogen peroxide (through an imino acid intermediate which can decompose spontaneously to the corresponding α-keto acid and NH_3_, Figure 4A; Pollegioni et al., 2008, 2013; Asano and Yasukawa, 2019). These enzymes have a wide variety of biomedical and biotechnological applications, including the production of different α-keto acids, important intermediate building blocks. Both LAAOs (EC 1.4.3.2, Pollegioni et al., 2013) and DAAOs (EC 1.4.3.3, Pollegioni and Molla, 2011) have been described. Inclusion of a catalase in AAO-based biotransformations is a common general strategy to avoid the toxicity of the H_2_O_2_ produced during the recycling of the FAD coenzyme necessary for AOO activity; thus, this AAO/catalase basic scaffold can be directly used for the KR of AA racemates, producing a mixture composed of 50% of enantiopure AA and 50% of the corresponding α-keto acid (Figure 4A; Pollegioni et al., 2008). Turner’s group proposed a preparative chemoenzymatic method for deracemization of NcAAs by inclusion of a non-selective chemical reductant, transforming back the intermediate imino acid produced by D- and L-AAOs till a racemic mixture of the original AA, thus allowing 100% conversion of the initial AA racemate (Figure 4A; Alexandre et al., 2002; Beard and Turner, 2002).

DAAO from porcine kidney, glutamic-pyruvic transaminase (E.C.2.6.1.2) from porcine heart, catalase from bovine liver and β-tyrosinase (tyrosine phenol lyase; EC 4.1.99.2) purified from *Citrobacter intermedius* were applied as a biosynthetic MEC for production of ^11^C-labeled L-Tyr and L-DOPA at the beginning of the 90’s, starting from racemic ^11^C-alanine (e.e. >98%, Figure 4B, Bjurling et al., 1990). A similar MEC was applied for the synthesis of ^14^C-L-Trp and 5-OH-Trp starting from racemic 1-^14^C- or 3-^14^C-alanine, replacing β-tyrosinase by tryptophanase (EC 4.1.99.1; Figure 4C, Pająk et al., 2018). Bienzymatic conversion of primary amines to NcAAs has been proposed using an engineered DAAO from porcine kidney and an R-enantioselective nitrilase (GenBank Acc. No AY487533, uncultured organism; Figure 4D). Moderate to low yields (62–73%) and e.e. (40%) were achieved with this methodology (Kawahara et al., 2017).

Despite the numerous KR applications proposed for LAAOs, their recombinant production in heterologous hosts has proven difficult (Pollegioni et al., 2013). Recent studies have greatly increased the knowledge on these enzymes (e.g., L- Trp-, L- Lys-, Gly- or L-Arg oxidases) which might boost the applications of LAAOs in the production of D-AA derivatives (Francis et al., 2017; Asano and Yasukawa, 2019). An L-Trp oxidase from *L. aerocolonigenes* has been successfully applied using two alternative chemoenzymatic MECs for the synthesis of different halogenated D-Trp derivatives (Figure 4E; Schnepel et al., 2019). In a first step, Trypthophan halogenase or Tryptophan synthase (TrpS), are used for the production of halogenated L-Trp derivatives. The L-enantiomer can then be stereo-destroyed by LAAO till the corresponding α-imino acid. Following the general use of non-selective chemical reductant applied for AAOs (Figure 4A), the imine intermediate can be transformed back till a racemic mixture of L- and D-α-AA. Whereas the D-α-AA will not be recognized by LAAO, the regenerated L-isomer enters back to the reaction, converting the system in a chemoenzymatic DKR (Figure 4E). Conversions achieved approximately 90%, with e.e. >92% (Schnepel et al., 2019). This MEC shows an interesting approach, since CLEAs obtained by cross-linking of precipitated tryptophan halogenase, flavin reductase and alcohol dehydrogenase were used, converting this crystalline precipitate into a multifunctional and recyclable MEC for the production of halogenated L-Trp derivatives in the gram scale (Frese and Sewald, 2015). Since Trypthophan halogenase/cofactor recycling CLEAs also recognized L-Trp, coupling of this system with an LAOO might use cheaper racemic mixtures of D,L-Trp as starting point.

Besides the above applications, AAOs are also pivotal enzymes in other MECs, and in fact, their use has been mainly linked to those applications; coupling of AAOs to amino acid dehydrogenases, ammonia-lyases or aminotransferases avoids the necessity of chemical transformation or further processing of the “undesired” α-keto acid produced by D- or L-AAO, allowing total conversion of the initial AA racemate till the corresponding D- or L-α-AA (see sections “Amino Acid Dehydrogenase-Based MECs,” “Ammonia Lyase-Based MECs,” and “Transaminase-Based MECs”).

### Amino Acid Dehydrogenase-Based MECs

**FIGURE 5:**
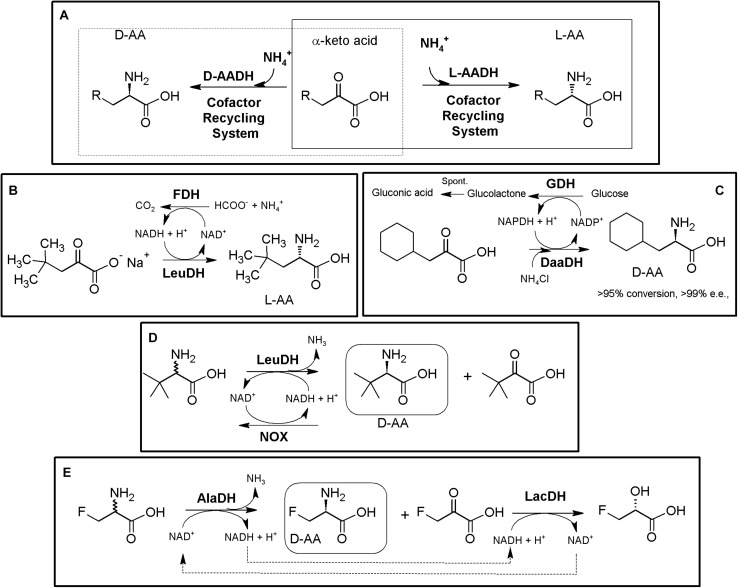
AADH-based multienzymatic cascades. **(A)** General scheme for the production of enantiopure AAs using AADH together an enzymatic cofactor-recycling system (e.g., FDH, GDH, NOX). D-system, dashed line; L-system, full line. The reactions are reversible, and can proceed in the other direction, although not indicated. **(B)** Production of D-*tert*-Leucine by kinetic resolution of racemic *tert*-leucine, employing L-leucine dehydrogenase from *Bacillus cereus* (LeuDH) and NADH oxidase from *Lactobacillus brevis* (NOX) for cofactor regeneration (Hummel et al., 2003). **(C)** Example on bioconversions carried out with L-AADH from *Bacillus stearothermophilus* and FDH from *Candida boidinii* (Krix et al., 1997). **(D)** Production of D-cyclohexylalanine in the gram scale. Engineered *meso*-2,6-D-diaminopimelic acid dehydrogenase from *Corynebacterium glutamicum* (DaaDH) and GDH as recycling system were used (Vedha-Peters et al., 2006). **(E)** Simultaneous synthesis of D-3-fluoroalanine (*S*-3-fluoroalanine; 60% yield, 88% e.e.) and L-fluorolactic acid starting from racemic 3-fluoroalanine using alanine dehydrogenase (AlaDH) coupled with lactate dehydrogenase (LacDH) (Gonçalves et al., 2000).

The reductive amination of α-keto acids to the corresponding α-AAs can be catalyzed (reversibly) by different NADH- (or NADPH-) amino acid dehydrogenases (AADHs; EC 1.4.1.X; Xue et al., 2018). From the operational point of view, high concentrations of ammonia are needed when using the reductive amination reaction, whereas the oxidative reaction yields α-keto acids when starting from amino acid substrates. Whereas most AADHs are L-enantioselective, D-AADHs (EC 1.4.99.1) have also been described (Vedha-Peters et al., 2006; De Wildeman et al., 2007; Hall and Bommarius, 2011; Li et al., 2012; Au et al., 2016; Akita et al., 2018; Zhang et al., 2019). This family of enzymes is greatly diverse, although in general, AADHs are promiscuous enzymes showing a high enantioselectivity (Xue et al., 2018). As way of example, Leucine dehydrogenase (LeuDH) and Phenylalanine dehydrogenase (PheDH) present broad substrate specificity; the former accepts hydrophobic, aliphatic, branched and unbranched or alicyclic keto acids, while the latter also accepts aromatic substrates (Hall and Bommarius, 2011). From a preparative perspective, nicotine amide coenzymes need to be recycled for continuous activity of AADHs, and thus, AADH-based systems precise of efficient coenzyme regeneration systems as a prerequisite for industrial processes. Coupling of L- or D-AADHs together with nicotinamide cofactor-recycling systems readily constitute bi- or multi-enzymatic modules for the production of enantiopure L-α- or D-α-AAs starting from α-keto acids (Figure 5A). As shown from the literature, after three decades this AADH-based MECs continue being of great relevance (Ohshima et al., 1989; Galkin et al., 1997; Krix et al., 1997; Bommarius et al., 1998; Patel, 2001; Menzel et al., 2004; Gröger et al., 2006; Cheng et al., 2016; Jiang and Fang, 2016; Chen et al., 2017; Liu et al., 2018; Luo et al., 2020).

Using the amination (reductive) reaction, the production of many different L-α-AAs has been reported. LeuDH from *Bacillus* species, together with FDH from *Candida boidnii* were partially purified and mixed for preparation of L-2-ABA, L-2-amino-3,3-dimethylpentanoic acid, L-2-Amino-5,5-dimethylhexanoic, L-2-Amino-4-ethylhexanoic acid, L-cyclohexyl-alanine, L-neopentyl-glycine or L-*tert*-leucine (e.e. > 99%). This system allowed production industrial scale of 30 kg of L-neopentyl-glycine in a 450 L reactor (Figure 5B, Krix et al., 1997). In an analogous process, Bristol-Myers Squibb produced 197 kg of L-allysine ethylene acetal using a 1600 L reactor, with dried cells containing PheDH together FDH (Liese et al., 2006). ^15^N-labeled norvaline and norleucine were produced combining LeuDH with a GDH/galactose mutarotase as cofactor recycling system to increase its effectivity (80–95% yield; Chiriac et al., 2008). On the other hand, different D-α-AAs have been obtained by AADH-based MECs (including ^13^C- and/or ^15^N-labeled DAAs; Vedha-Peters et al., 2006; Akita et al., 2018). Coupling of engineered *meso*-diaminopimelate dehydrogenase (EC 1.4.1.16) together with a GDH recycling system allowed the production of more than 20 different D-α-AAs (e.e. 95 to >99%, except for alanine, e.e. 77%; the later result was possibly due to the presence on an alanine racemase in the cellular extracts used; Vedha-Peters et al., 2006; Akita et al., 2018). This system was used for the gram scale synthesis of D-cyclohexylalanine (Figure 5C).

Taking advantage of the deamination (oxidative) reaction, kinetic resolution of D,L-*tert*-Leucine was achieved by coupling L-LeuDH with a highly efficient irreversible NOX allowing D-*tert*-Leucine production (e.e. > 99%). The corresponding α-keto acid was also obtained as by-product of the reaction (Figure 5D) (Hummel et al., 2003). Alanine dehydrogenase coupled with lactate dehydrogenase and internal cofactor regeneration were applied for the simultaneous synthesis of *S*-3-fluoroalanine (D-3-fluoroalanine; 60% yield, 88% e.e.) and L-fluorolactic acid starting from racemic 3-fluoroalanine (Figure 5E, Gonçalves et al., 2000).

**FIGURE 6:**
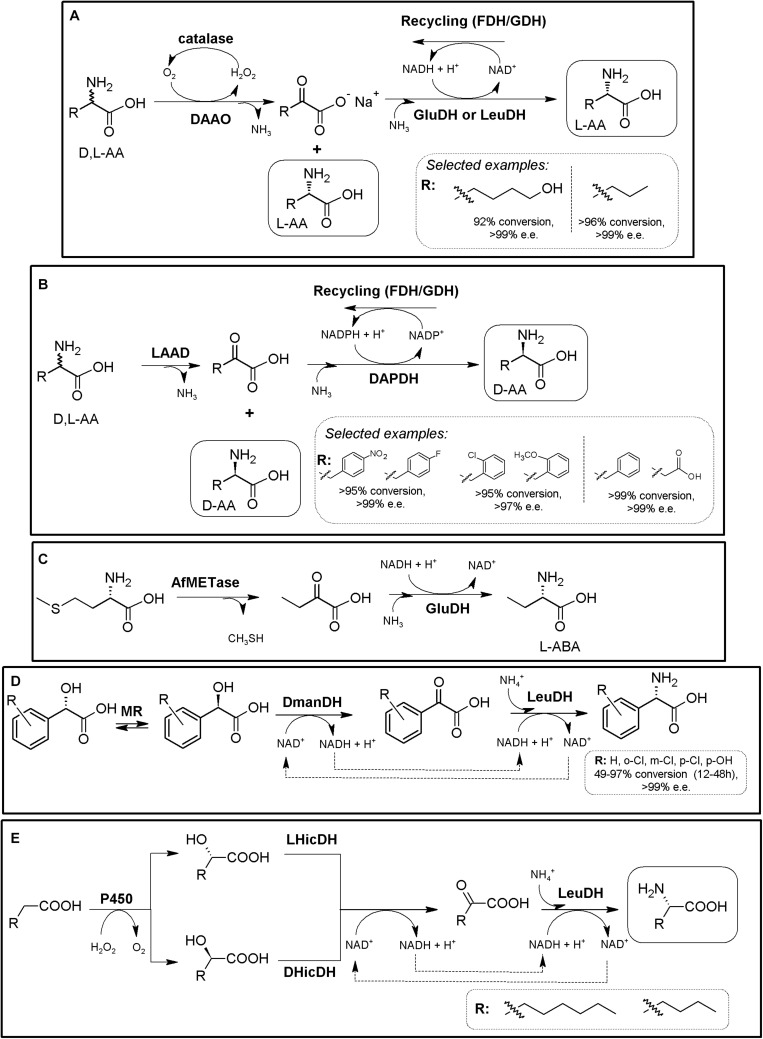
Additional examples on the production of D- or L-AAs using AADH-based MECs. **(A)** General scheme for the conversion of racemic AAs till the corresponding enantiopure L-α-amino acids using a DAOO-catalase/AADH-recycling system MECs. Selected examples were extracted from Patel (2001) (left) and Qi et al. (2017) (right), respectively. **(B)** General scheme for the conversion of racemic AAs till the corresponding enantio-enriched or enantipure D-α-AAs. L-AAD, WT or (engineered enantioselective meso-diaminopimelate dehydrogenase (DAPDH) and a cofactor-recycling system (e.g., FDH or GDH) were applied. Selected examples were extracted from Parmeggiani et al. (2016) (left) and Zhang et al. (2019) (right). **(C)** Conversion of L-methionine into L-ABA using immobilized L-methioninase (AfMETase) together with glutamate dehydrogenase (GluDH) on polyacrylamide and chitosan (El-Sayed et al., 2015). **(D)** Deracemization of mandelic acid derivatives to enantiopure L-PheGly derivatives via a redox-neutral biocatalytic MEC. Mandelate racemase (MR), D-mandelate dehydrogenase (DmanDH) and LeuDH were coupled to synthesize different PheGly derivatives (Resch et al., 2010). **(E)** MEC for the enantioselective α-amination of fatty acids to α-AAs. Combination of P450 peroxygenase from *Clostridium acetobutylicum* (P450), two stereocomplementary 2-hydroxyisocaproate dehydrogenase from *Lactobacillus confusus* (LHicDH) and *Lactobacillus casei* (DHicDH) and LeuDH from *Bacillus cereus* allowed partial conversion of hexanoic acid to L-norleucine (e.e. > 97%; Dennig et al., 2018).)

The above-described systems can be expanded with the inclusion of biocatalysts allowing the production of the α-keto acid substrates from other low-cost materials, such as AAOs (section “Amino Acid Oxidase-Based MECs”), threonine deaminase (ThrD^3^, threonine ammonia lyase) or “real” amino acid deaminases. L-amino acid deaminases (L-AADs, EC 1.4.99.B3, Molla et al., 2017; Melis et al., 2018; Nshimiyimana et al., 2019) have been described as membrane-bound cytochrome-like flavoenzymes that catalyze the oxidative deamination of different AAs for the formation of their corresponding α-keto acids and ammonia; they have gained interest in the last lustrum since they can replace LAAOs in biotechnological applications (Molla et al., 2017). Both AAOs and AAD can thus be engaged to enantiocomplementary AADHs, for the theoretical production of L-α-AAs (DAAO/catalase/L-AADH/cofactor recycling) or D-α-AAs [(LAAO/catalase) (or LAAD)/D-AADH/cofactor recycling]. As way of example, L-6-hydroxyleucine was produced using a MEC comprising two enzymatic modules (Figure 6A, DAOO-catalase and AADH-recycling system; Patel, 2001 and references therein). L-norvaline has been also produced following this strategy, using DAAO/LeuDH/catalase/FDH (Figure 6A, Qi et al., 2017).

L- and D-ABA have received huge attention in the literature, and has been produced in many different reports using ThrD. As way of example, ThrD was coupled with LeuDH and GDH or FDH cofactor-recycling systems (Tao et al., 2014). A scale-up of the process (30 L of reaction in a 50-L fermenter) allowed the production of 29.2 mol L-ABA (97.3% theoretical yield), with a productivity of 6.9 g⋅L^–1^⋅h^–1^ (Tao et al., 2014). This MEC module has also been used to generate a heterologous biosynthetic pathway leading to the production of L-ABA in *Saccharomyces cerevisiae* (Weber et al., 2017). Metabolic engineering allowed the expansion of the latter system for the production of *S*-2-aminobutanol. D-ABA was produced by a tri-enzymatic cascade with cell-free extract or purified enzymes, composed of ThrD, D-amino acid dehydrogenase and FDH, starting from L-Thr (>95% yield and >99% e.e.; Chen et al., 2017). More than 15 different enantio-enriched and enantiopure D-α-AAS -including several D-phenylalanine derivatives- have been produced coupling a “real” L-AAD to engineered enantioselective meso-diaminopimelate dehydrogenase and a cofactor-recycling system (conversions from 45.3 to >99%, e.e. values ranging 52.1 to >99%; Figure 6B, Parmeggiani et al., 2016; Zhang et al., 2019). Racemic mixtures might be used as well as pure L-AAs, turning it into a more efficient and cheaper system. A scarcely described strategy utilized immobilized PLP-dependent L-methioninase (L-methionine γ-lyase, EC 4.4.1.11) together with GluDH on polyacrylamide and chitosan (with no regeneration system) for the production of L-ABA, starting from L-methionine (Figure 6C, El-Sayed et al., 2015).

An alternative strategy allowing *in situ* production of α-keto acids for further conversion by AADHs consist in the oxidation of α-hydroxy acids. This is the case of the MEC combining mandelate racemase, mandelate dehydrogenase and L-AADHs, allowing the production of L-phenyglycine from mandelic acid; this system further uses an elegant internal cofactor recycling system (Resch et al., 2010). This 3-step one-pot reaction has been efficiently applied in a whole cell system, reaching a production of 79.70 g⋅L^–1^⋅d^–1^ (Tang et al., 2020). It was also used for the production of different L-PheGly derivatives starting from different mandelic acid derivatives; conversions ranging 49–97% were achieved, with e.e., >97% (Figure 6D, Fan et al., 2015). Further expansion of this system allows *in situ* production of an α-hydroxy acid starting from fatty acids. This strategy was shown for the production of L-norleucine starting from hexanoic acid. This MEC consisted in a combination of a P450 peroxygenase, two stereocomplementary L- and D-hydroxyisocaproate dehydrogenases along with LeuDH; no extra recycling system was needed, since this MEC also provide internal cofactor regeneration (Figure 6E). L-norleucine conversion was lower than 35% with up to 5 mM substrate concentrations; e.e. > 97%; L-ABA production was also reported (Dennig et al., 2018).

### Ammonia Lyase-Based MECs

Ammonia-lyases (ALs, EC 4.3.1.X, defined as carbon-nitrogen lyases that release ammonia as one of the products) comprise a heterogenous enzymatic group catalyzing the reversible cleavage of C-N bonds, typically of α-AAs, producing an unsaturated (or cyclic) derivative and ammonia. More than 30 different EC subclasses of ALs are reported, showing remarkable structural, functional and mechanistic differences, which can be broadly grouped into seven main classes (Parmeggiani et al., 2018; Viola, 2020). Besides of the interest on ALs for the production of β-AAs and other APIs (Sariaslani, 2007; Turner, 2011; Xue et al., 2018), applications for the synthesis of NcAAS have also been reported, being aromatic amino acid ALs [phenylalanine AL (PAL), histidine AL (HAL) and tyrosine AL (TAL)] the most relevant and studied enzymes, showing a marked L-enantioselectivity (Turner, 2011; Parmeggiani et al., 2015; Zhu et al., 2019). Although as an isolated case, the acyclic amino acid propargylglycine was recognized by a PAL enzyme (Weiser et al., 2015).

**FIGURE 7:**
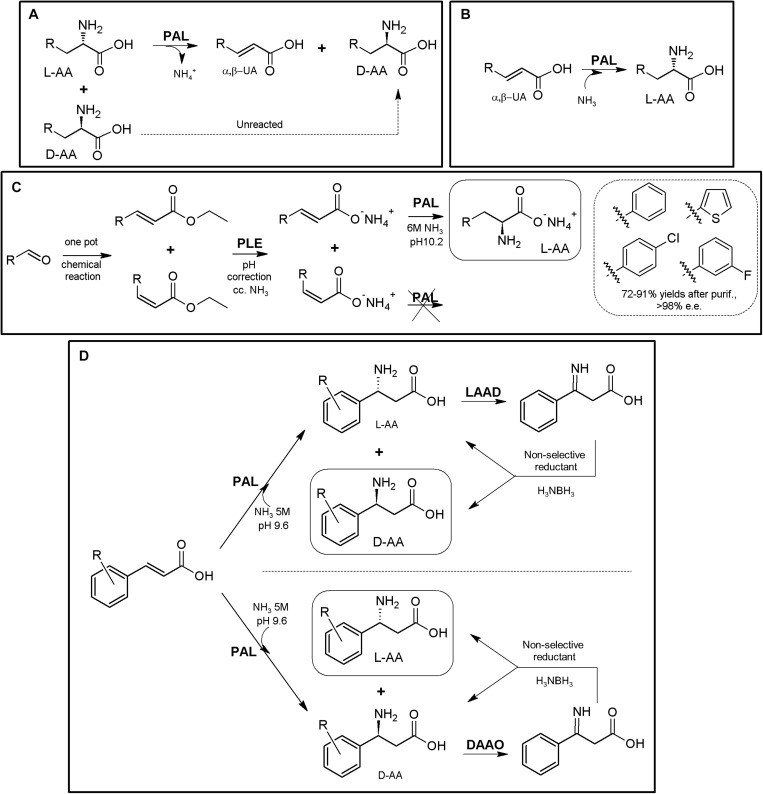
AL-based production of α-AAs. **(A)** General scheme of PAL-mediated deracemization of racemic mixtures of AAs to enantio-enriched or enantiopure D-α-AAs mediated by deamination of L-α-AAs. **(B)** Asymmetric amination of α,β-unsaturated acids for the production of enantio-enriched or enantiopure L-α-AAs. α,β-unsaturated acid (α,β-UA) (Tork et al., 2019). **(C)** Production of L-Phe and other L-α-NcAAs using a chemoenzymatic approach. The starting arylaldehydes were transformed into the corresponding α,β-unsaturated acids by the Wittig reaction; further application of porcine liver esterase (PLE), PAL from parsley and ammonia allowed transformation into L- phenyl-, L-4- chlorophenyl-, L-3-fluorophenyl- or L-thiophen-2-yl-alanine (Paizs et al., 2006). **(D)** Chemoenzymatic synthesis of L- and D-phenylalanine derivatives using PAL/LAAD and PAL/DAOO systems (Parmeggiani et al., 2018).

As for other enzymes, their reversible catalytic properties together with their enantioselective character allows the use of isolated ALs both for (i) KR processes for deracemization of AAs (obtaining a mixture of D-α-AAs and α,β-unsaturated acid, Figure 7A, Poppe and Retey, 2003; Turner, 2011; Tork et al., 2019) or (ii) asymmetric synthesis of L-α-AAs (Figure 7B), using high concentration of ammonia to shift PAL equilibrium toward the amination reaction starting from achiral α,β-unsaturated acids (Poppe et al., 2012; Tork et al., 2019). Other outstanding feature is the non-necessity of expensive cofactors or recycling systems, thus providing a cost-effective and easier application. It is important to highlight that aminomutase-like activity has been detected in PALs (Weise et al., 2015, 2018); whereas this fact might be a drawback for general application in the synthesis of enantio-enriched α-AAs, it might also open up new biotechnological properties of PALs.

Enantiopure L-arylalanines were produced by one-pot chemoenzymatic reaction using porcine liver esterase and PAL from parsley and ammonia (Figure 7C). The starting materials were arylaldehydes, which were synthesized *in situ* from the corresponding α,β-unsaturated acids by the Wittig reaction (Paizs et al., 2006). Coupling of PAL together with LAAD allowed the conversion of cinnamic acid derivatives into the corresponding enantio-enriched D-α-AA by a chemoenzymatic process (Figure 7D) using *in situ* non-selective reductants (conversions 62–80%; e.e. values 98% to >99%; Parmeggiani et al., 2015; conversions 12–96%; e.e. values 72% to >99%; Zhu et al., 2019). Although PAL is reported to be mainly L-enantioselective, PAL-catalyzed amination of the cinnamic acids were reported to lead to the formation of significant levels of the D-enantiomer^4^ (in particular, for cinnamic acids with an electron-deficient aromatic ring; Parmeggiani et al., 2015). Taking advantage of the unexpected production of both enantiomers over the time, coupling of this system with a DAAO, together with the use of a non-selective reductant, produced different L-α-AAs starting from α,β-unsaturated acids (conversions 66–82%; e.e. values > 99%; Parmeggiani et al., 2015; Figure 7D).

Labeling of L-Tyr with carbon and hydrogen isotopes was achieved by coupling of PAL and L-phenylalanine 4′aminooxygenase (Pająk et al., 2018); this strategy might be expanded for the production of L-DOPA using p-hydroxyphenylacetate 3-hydroxylase (Min et al., 2015), including tetrahydropterin- and NADH-recycling systems (Hara and Kino, 2013) (Supplementary Figure S4).

### Transaminase-Based MECs

**FIGURE 8:**
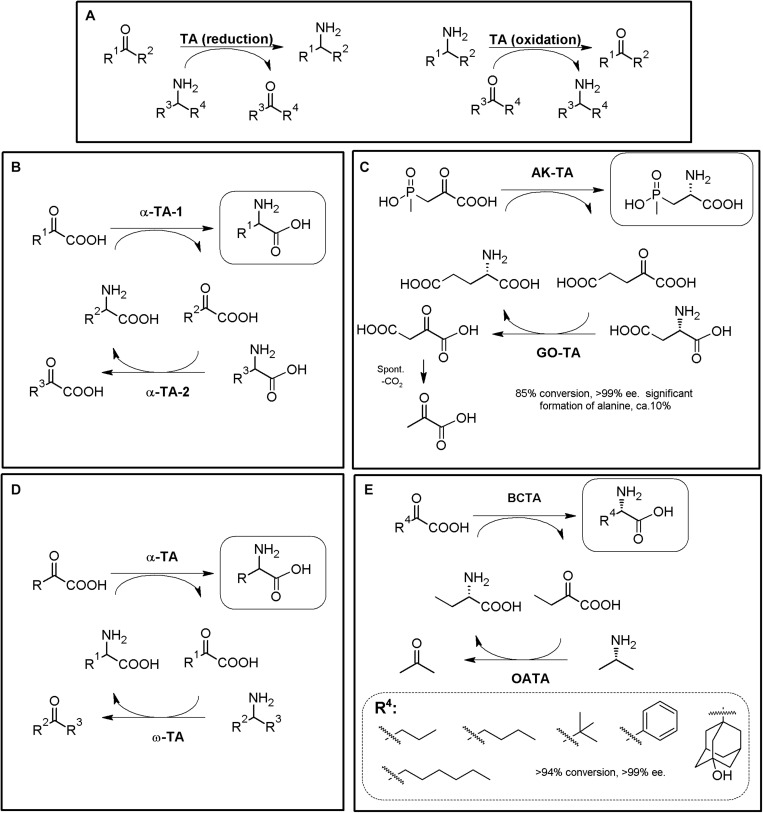
TA-based production of α-AAs. **(A)** General scheme for reversible α-TA and ω-TA catalysis. When *R*^2^ = COOH, the reaction correspond to an α-TA; ω-TA do not necessarily precise a carboxylic moiety in this position, although they usually also recognize α-AAs as substrates. Both the direct reaction (reduction, dashed line) and the reverse reaction (oxidation, full line) can be used for the production of enantiopure AAs (i.e., starting from keto/keto acids or from amines/AAs). **(B)** General scheme for production of enantiopure or enantio-enriched α-AAs using two different α-TAs. The selected example **(C)** consists on the enzymatic synthesis of the herbicide L-phosphinothricin using 4-aminobutyrate:2-ketoglutarate (AK-TA) transaminase from *E. coli* and glutamate:oxalacetate transaminase (GO-TA) from *Bacillus stearothermophilus* (24 h reaction, 0.5M substrate concentration). L-Glu is used as the amino donor for AK-TA, and transformed to a-ketoglutarate; the latter compound is transformed back to L-Glu by GO-TA, which uses L-Asp as amino donor and converts it to oxaloacetic acid. Oxaloacetic acid decarboxylate spontaneously to pyruvate, driving the AK-TA reaction till the product of the reaction (Bartsch et al., 1996; Ricca et al., 2011). **(D)** General scheme for the production of enantiopure α-AAs using α-TA/ω-TA combination. The selected example **(E)** consists on the enzymatic synthesis of different L-α-AAs using *S*-selective branched-chain transaminase (BCTA) from *E. coli* and ω-TA from *Ochrobactrum anthropi* (OATA) (Park et al., 2013b).

Transaminases (TAs, EC 2.6.1.X, also known as aminotransferases) are a heterogeneous group of enzymes catalyzing the transfer of an amino group between two different molecules. In this kind of enzymes, different enantioselective PLP-dependent TAs catalyze the (reversible) transfer of an amino group between an amino donor and an amino acceptor (in general, a carbonyl group such as α-ketocarboxylic acids or ketones), yielding chiral amines with a new stereocenter (e.g., AAs, Figure 8A, Guo and Berglund, 2017). During TA catalysis, PLP-recycling is accomplished, which is an advantage in enzymatic synthesis since no additional cofactor-recycling system is needed (Figure 8A). TAs are well documented enzymes for the synthesis of NcAAs (Meiwes et al., 1997; Taylor et al., 1998; Li et al., 2002; Rozzell and Bommarius, 2002), but their biotechnological interest is far beyond the production of these compounds, since they allow the production of other important molecules [e.g., β-AAs (Rudat et al., 2012) or amines (Höhne and Bornscheuer, 2009)]. TAs continue receiving huge attention, and have been reviewed extensively during the last decade due to their huge biotechnological interest (e.g., Mathew and Yun, 2012; Simon et al., 2014; Guo and Berglund, 2017; Slabu et al., 2017; Patil et al., 2018; Xue et al., 2018; Cutlan et al., 2019). Protein engineering strategies have been broadly used to evolve ω-TAs (e.g., Park et al., 2013a, b; Walton et al., 2017), and a database on sequences and structures of biotechnologically relevant engineered ω-TAs is available (Buß et al., 2018).

TAs^5^ with potential application in α-NcAA production can be broadly grouped according to the position of the transferred amine group with respect to the carboxylic moiety (when the substrate/reaction product is an AA). α-TAs catalyze the transfer of the amino group at the α-carbon, whereas ω-TAs (also referred to as amine-TAs) transfer the amino group to a carbon further away from the carboxylic group^5^ (Guo and Berglund, 2017; Slabu et al., 2017). Both *R*- and *S*-enantioselective and promiscuous TAs have been reported (as natural or engineered enzymes, Taylor et al., 1998; Koszelewski et al., 2010); a unique L to D-stereoinverting hydroxyphenylglycine aminotransferase has also been reported (Müller et al., 2006; Walton et al., 2017). Since the enantioselectivity of TAs can be greatly affected by the reaction conditions, enantio-enriched compounds might be produced in some cases (Koszelewski et al., 2010); TAs are also described to suffer substrate and/or product inhibition (Guo and Berglund, 2017), and produce by-products which need to be eliminated or separated; these aspects need to be taken into account when designing TA-based MECs. Since shifting the reaction equilibrium to the desired product of the reaction is required to maximize the productivity of TAs, different strategies have been already proposed to overcome some of TAs potential drawbacks (e.g., distillation, use of biphasic reaction systems, recycling of the carbonyl compounds, degradation/transformation of by-products of the reaction (Koszelewski et al., 2010; Guo and Berglund, 2017; Patil et al., 2018). As way of example, enzymatic methods to remove pyruvate from the reaction medium (if alanine is used as amino donor) can be accomplished with acetolactate synthase, lactate dehydrogenase reduction (together a nicotinamide recycling system such as FDH, GDH, or NOX), pyruvate decarboxylase (or phenyl pyruvate decarboxylase with phenyl alanine as amine donor) or alanine dehydrogenase (Koszelewski et al., 2010; Simon et al., 2014).

One of the enzymatic combinations that can be carried out for shifting TA reaction equilibrium accounts on coupling two different TAs in a one-pot two-step procedure (Figures 8B–E). In this general strategy, a “primary” TA converts an α-keto acid to the corresponding α-AA. The “secondary” TA (in general a different α-TA or a ω-TA) transforms the α-keto acid back, replenishing the initial amino donor (Figures 8B,D) (Taylor et al., 1998; Ager et al., 2001; Li et al., 2002; Gefflaut et al., 2012; Park et al., 2013a). The enantiomer obtained in these systems will depend on the enantioselectivity of the TAs used, and thus, both D- or L-α-AAs can be obtained; the use of ω-TAs in these systems presents many advantages, since the secondary reaction can be conducted using amino donors different to AAs, reducing the number of possible interferences among the reactivity of both TAs. Enzymatic synthesis of the herbicide L-phosphinothricin was achieved by using 4-aminobutyrate:2-ketoglutarate transaminase from *E. coli* and glutamate:oxalacetate transaminase from *Bacillus stearothermophilus* (Figure 8C, 24 h reaction, 0.5M substrate concentration; Bartsch et al., 1996; Ricca et al., 2011). Different L-α-AAs were obtained by using *S*-selective branched-chain transaminase from *E. coli* and ω-TA from *Ochrobactrum anthropi* (Figure 8E, Park et al., 2013a).

**FIGURE 9:**
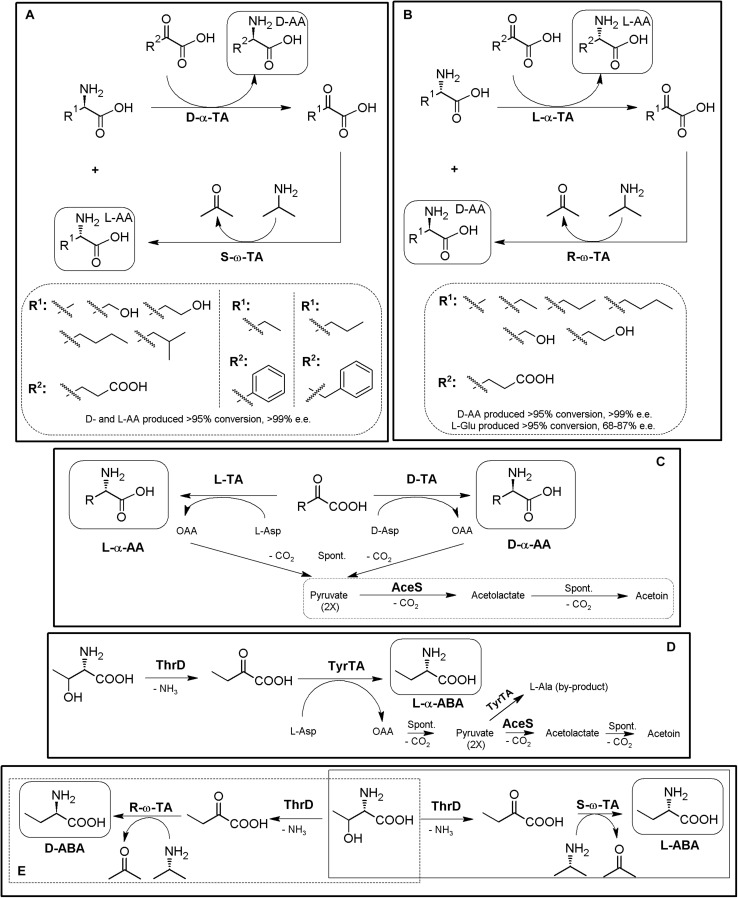
Additional examples on TA-based MECs. **(A)** General scheme for the coupling of enantiocomplementary D-α-TA/S-ω-TA for deracemization of AA mixtures, yielding two different enantiopure α-AAs of opposite chirality. Selected examples for the production of L-α-AAs (*R*^1^) and D-α-AAS (*R*^2^) produced by D-amino acid transaminase from *Bacillus sphaericus* (D-α-TA) and *S*-selective-ω-TA *Ochrobactrum anthropi* or *Paracoccus denitrificans* (S-ω-TA) (Park and Shin, 2014). **(B)** Coupling of enantiocomplementary L-α-TA/R-ω-TA for deracemization of AA mixtures, yielding two different enantiopure/enantioenriched α-AAs of opposite chirality. Selected examples for the production of L-α-AAs (*R*^1^) and D-Glu (*R*^2^) by branched-chain TA from *Escherichia coli* (L-α-TA) and an engineered variant of (R)-selective ω-TA from *Arthrobacter* sp (R-ω-TA) (Park and Shin, 2014). **(C)** General scheme for the preparation of different D- or L-α-NcAAs using D-TAs or L-TAs coupled to acetolactate synthase (AceS). D- or L-Asp are used as amino donors, producing oxaloacetate (OAA) as by-product of the reaction. OAA decarboxylate spontaneously to pyruvate, which is converted to acetolactate by AceS, followed by spontaneous decarboxylation to acetoin (AceS reaction sequence inside a dashed box). AceS thus allows shifting the reaction toward the acetoin, and also prevents pyruvate from being aminated to L-alanine (Taylor et al., 1998). **(D)** Preparation of L-ABA in whole cell *E. coli* system starting from L-Thr. The system comprised threonine deaminase from *E. coli* (ThrD), aromatic TA from *E. coli* (TyrTA), and AceS. Starting from 0.5 M of L-Thr and L-Asp, concentrations of the products of the reaction after 24 h were 27.71 mg⋅mL^– 1^ (L-ABA), 1.23 mg⋅mL^– 1^ (L-Ala) and 1.01 mg⋅mL^– 1^ (L-Asp) (Fotheringham et al., 1999). **(E)** General scheme for the biosynthesis of L-ABA (continuous line) or D-ABA (dashed line) using TD*/ω-TA systems (Park et al., 2013a).

Since α/α- or α/ω-TA MECs with different substrate specificities and different amino donors/acceptors combinations are possible, concomitant production of different enantiopure compounds can be achieved using TA-based MECs (Li et al., 2002; Wenda et al., 2011; Park et al., 2013a, b). Thus, if racemic mixtures of AAs are used as the reactive of the reaction instead of α-keto acids, one-pot production of two different enantiopure (or enantioenriched) compounds of opposite chirality can be achieved (Figures 9A,B) (Cho et al., 2003; Park and Shin, 2014, 2015). An enantiocomplementary D-α-TA/S-ω-TA system allowed conversions over 95% with e.e. >99% for different AA pairs (Figure 9A). On the other hand, a L-α-TA/R-ω-TA system was effective for the production of enantiopure D-α-AAs (>95% conversion, >99% e.e.), although the L-α-AA obtained in the reaction presented a lower enantiopurity (L-Glu, 68–87% e.e; Figure 9B) (Park and Shin, 2014). Simultaneous synthesis of (*S*)-AAs (Phe and ABA) and (*R*)-amines was also conducted using different α/ω-TA systems in a two-liquid phase system to avoid product inhibition by removing it to the organic phase. Using 0.3 M of 2-oxobutyrate and 0.3 M of racemic-methylbenzylamine, 276 mM of (*S*)-2-aminobutyrate (>99% e.e.) and 144 mM of (*R*)-methylbenzylamine (>96% e.e.) were produced in 9 h (Cho et al., 2003).

As mentioned above, shifting of α-TAs reaction equilibrium can be achieved by coupling with enzymes allowing the degradation/transformation of the by-products of degradation (e.g., pyruvate if alanine is used as amino donor). The use of acetolactate synthase (AceS) was one of the first proposed strategies to remove the by-products of the reaction, by condensation of two pyruvate molecules to acetolactate, which spontaneous decarboxylates to acetoin (Figure 9C) (Fotheringham et al., 1998). α-TA/AceS system also allowed the production of NcAAs such as L-*tert*-Leu or L- and D-ABA or D-Glu (Fotheringham et al., 1998; Taylor et al., 1998). Since α-keto acids can be the substrates of TAs (Figures 8, 9), enzymatic conversion of different compounds till the corresponding α-keto acids constitute a general strategy to expand TA-based MECs; AAOs, AADH or deaminases (sections “Amino Acid Oxidase-Based MECs” and “Amino Acid Dehydrogenase-Based MECs”) are thus candidates to be coupled with TAs for utilization of AAs as initial substrates. Inclusion of L-amino acid deaminases^6^ into the α-TA/AceS system allows the synthesis of different AAs, depending on the enantioselectivity of the enzymes. As way of example, L-ABA was obtained from L-Thr; whole-cell systems containing aromatic TA, ThrD and AceS were used efficiently for the production of L-ABA, although L-Ala was obtained as a by-product of the reaction (pyruvate is formed by spontaneous decomposition of oxaloacetate during the reaction, and it can be aminated back to L-Ala if not removed from the reaction) (Figure 9D; Fotheringham et al., 1999; Ager et al., 2001). In an analogous approach, NSC Technologies (Monsanto) produced different D-α-AAs using D-aminotransferase from *Bacillus* sp. and AceS, with a capacity in the multiton⋅a^–1^ scale (Liese et al., 2006).

Removal of L-Ala by-product formed by TA as a result of secondary amination of pyruvate formed by decomposition of OAA (Figure 9D) was possible by coupling the ThrD/TyrTA/AceS MEC with DAOO and alanine racemase (Supplementary Figure S2). This expanded system also allowed the production of L-ABA, lowering the concentrations of L-alanine by-product in the reaction (Zhu et al., 2011). This five enzyme-MEC allowed a large scale-preparation of L-ABA by combining in a whole cell system (reaction volume 1500 L in a 2000-L jar fermenter), resulting in an L-ABA concentration of 25.38 g⋅L^–1^ (246 mM) at the second stage of the reaction (Zhu et al., 2011).

Most of the problems arisen from these initial TA systems (such as secondary amination of the α-keto acids produced during the reaction, e.g., pyruvate) can be generally overcome by substitution of α-TA by ω-TA. Simplification of previous multienzymatic systems utilized for the synthesis of ABA (Figures 9C,D and Supplementary Figure S2) was achieved by using a ThrD together a ω-TA. With this configuration, both L- and D-ABA were synthesized by using S- or R-ω-TA, respectively. L-Thr (0.3 M) was transformed in less than 24 h, with conversions over 99% and e.e. > 99% (Figure 9E, Park et al., 2010, 2013b). A similar system allowed for concomitant production of D-Thr and D- or L-ABA starting from racemic Thr (>99% e.e.; Han, and Shin, 2015). An alternative approach for the production of D- or L-ABA consisted in coupling an L-methioninase with a D-TA from *Bacillus* sp or an L-TA from *E. coli*, using methionine as substrate (Silva et al., 2019).

**FIGURE 10:**
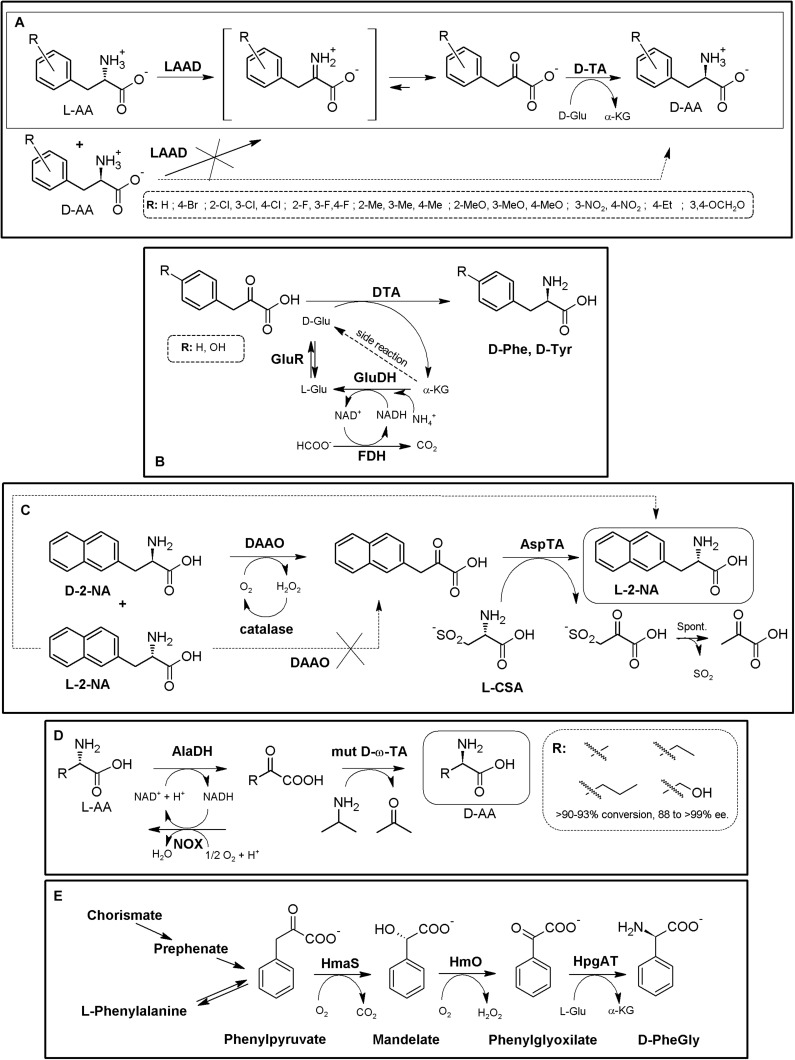
Additional examples on TA-based MECs. **(A)** Biocatalytic cascade for the asymmetric synthesis of D-Phe derivatives starting from L-α-AAs (solid square) or from α-AA racemates (dashed square). L-amino acid deaminase (LAAD) and engineered T242G mutant D-amino acid aminotransferase (D-TA) were used. (After 4 h of reaction e.e. values ranging 90 to >99% were achieved (Walton et al., 2017). **(B)** Production of D-Phe and D-Tyr using D-TA from *Bacillus* sp. YM-1 (D-TA) coupled to *in situ* regeneration/production of the amino donor of the reaction by [GluDH/FDH/Glutamate Racemase (GluR)] (Bae et al., 1999). **(C)** Multi-step enzyme catalyzed deracemization of D,L-2-naphthylalanine (2-NA) using DAAO/catalase/TA MEC (98% yield and 99.5% e.e.). *Lactate dehydrogenase was used to monitor the reaction course, although its inclusion in the system might help to avoid pyruvate transforming till L-Ala (Caligiuri et al., 2006). **(D)** Conversion of racemic AAs into D-α-AAs using AlaDH, NOX and engineered ω-TA (mut D-ω-TA) MEC (Han and Shin, 2018). **(E)** Biosynthetic pathway for D-PheGly production. Whole cells of *E. coli* were used, overexpressing hydroxymandelate synthase (HmaS) and hydroxymandelate oxidase (Hmo) (both from *A. orientalis* or *S. coelicolor*) and D-(4-hydroxy)-phenylglycine aminotransferase from *Pseudomonas putida* (HpgAT) (Müller et al., 2006).)

Recently, a “real” promiscuous LAAD from *Proteus mirabilis* together a engineered D-TA from *Bacillus* sp. YM-1 has been applied for the synthesis of different D-Phe derivatives starting from L- or D,L-α-AAs using whole cells (Figure 10A, Walton et al., 2017). Besides these derivatives, the authors reported that the D-TA variants shown in this work also displayed increased activity for D-α-AAs with aliphatic or polar side chains. Furthermore, they also suggested that the disadvantage of using D-Glu as amino donor could be overcome by generating the donor substrate *in situ* with Glu- or Asp-racemase and by replacing LAAD from *Proteus mirabilis* with that from *Proteus myxofaciens*, shown to not deaminate L-Glu or L-Asp (Walton et al., 2017). Amino acid racemases have been included for *in situ* regeneration/production of the amino donor of the TA reaction. As way of example, D-TA coupled to GluDH-FDH-glutamate racemase recycling system allowed the production of D-Phe or D-Tyr (48 and 60 g⋅L^–1^, respectively. e.e. > 99%, 35 h) from the corresponding α-keto acids (Figure 10B, Bae et al., 1999, 2002). If amino acid racemases are included in these MECs, it is advisable to know whether the AR can recognize the product of the reaction.

AAOs have also been coupled with TAs to obtain different NcAAs. DAAO/catalase, together with an L-aspartate amino transferase (α-TA) were used to produce L-2-naphthyl alanine with a 98% yield and 99.5% e.e. (Figure 10C, Caligiuri et al., 2006). The use of L-CSA as amino donor allows shifting of the reaction by spontaneous degradation of the thio-α-keto acid to pyruvate. A NADH-dependent lactate dehydrogenase was added in this system to transform the produced pyruvate, allowing to follow the course of the reaction spectrophotometrically; on the other hand, addition of this enzyme could also become a general idea to remove the pyruvate formed, avoiding putative transformation to L-Ala by α-TA (see above). An alternative strategy was proposed afterward by inclusion of a LAAO together a D-TA, using D-aspartate as the amino donor, which can be generated with an aspartate racemase (Tessaro et al., 2008). L-ABA was produced using an engineered DAAO/catalase module together with ω-TA (Seo et al., 2012). Together with the ω-TA from *Vibrio fluvialis* JS17, a fusion protein was created with DAAO from *Rhodotorula gracilis* and *Vitreoscilla* hemoglobin, since it was earlier reported to significantly enhance the DAAO activity in the production of D-fluoroalanine. Using whole cells together with a biphasic system (to partly overcome ω-TA inhibition by benzaldehyde), 500 mM racemic ABA were transformed to 485 mM L-homoalanine (>99% e.e.; Seo et al., 2012). Racemic glufosinate was also converted to the L-enantiomer by application of a DAAO and different TAs (Green and Gradley, 2017).

When coupled to TAs, AADHs also can provide a deracemization process starting from AA racemates. An L-AlaDH/NOX system, together with an engineered D-ω-TA from *Arthrobacter* sp. was shown as an effective MEC for deracemization of AA racemates (Figure 10D). Synthesis of D-alanine with a 95% conversion yield (starting form 100 mM solutions) and >99% e.e. was achieved after 24 h (Han and Shin, 2018). Coupled D-TA and L-PheDH from *Lysinibacillus sphaericus* (together with a recycling system using ethanol and alcohol dehydrogenase) were applied for the production of different *para-*halogenated derivatives (Br-, Cl-, and F-) of Phe, as well as Tyr, *via* stereo-inversion of the D-enantiomers to the L-isomer; e.e. > 99% (Khorsand et al., 2017).

Recently, coupling of different enantioselective SAM-dependent α-keto acid methyltransferases (MT) with a halide methyltransferase (HMT) and a ω- or L-α-TA has been successfully applied for the synthesis of several enantio-enriched D- and L-β-methyl-α-AAs (Supplementary Figure S3, Liao and Seebeck, 2020). An *S*-adenosylhomocysteine nucleosidase-deficient *E. coli* strain was necessary for this approach, using CH_3_I as the alkylating agent. Conversions ranging 39 to >95% were achieved for different substrates. Methylation resulted in a high stereoselectivity, with 3R:3S methyl ratios ranging 92:8 to 99:1.

**FIGURE 11:**
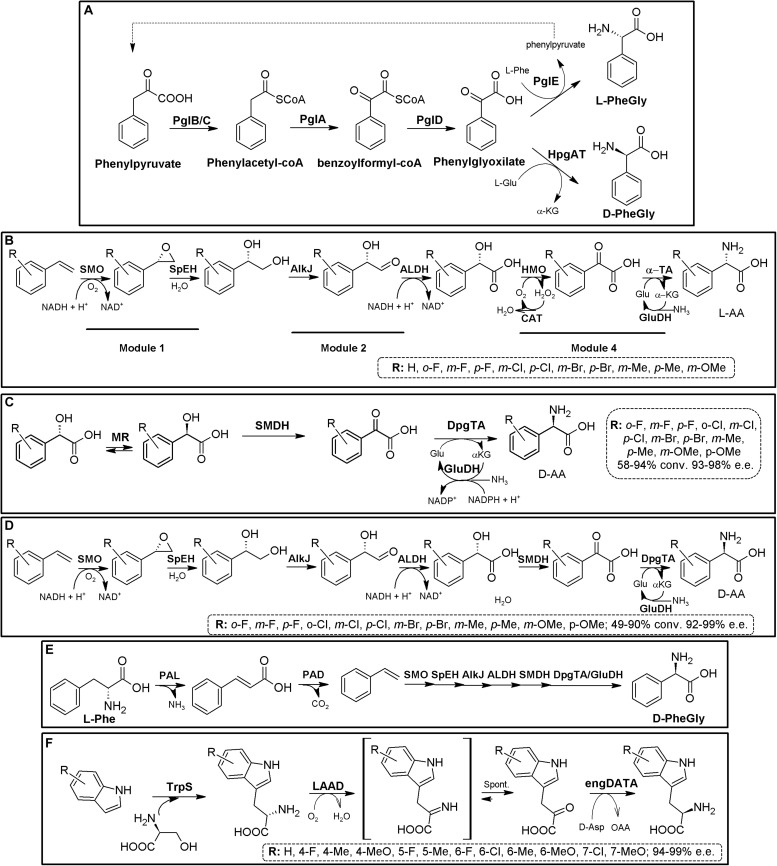
Additional examples on TA-based MECs. **(A)** Schematic representation of the natural L-PheGly biosynthetic pathway from *S. pristinaespiralis* (*lpg* operon, pglA-E). PglA, PheGly dehydrogenase. PglB, Pyruvate dehydrogenase α-subunit. PglC, pyruvate dehydrogenase β-subunit. PglD, thioesterase. PglE, L-PheGly aminotransferase. Inclusion of the stereoinverting D-(4-hydroxy)-PheGly aminotransferase from *Pseudomonas putida* (HpgAT) allowed the production of D-PheGly (Moosmann et al., 2020). **(B)** Conversion of styrenes to L-PheGly derivatives with *E. coli* multimodular systems. SMO, styrene monooxygenase from *Pseudomonas* sp. VLB120; SpEH, epoxide hydrolase from *Sphingomonas* sp. HXN-200; AlkJ, alcohol dehydrogenase from *P. putida* GPo1; ALDH, phenylacetaldehyde dehydrogenase from *E. coli*; HMO, hydroxymandelate oxidase from *S. coelicolor* A3(2); CAT, catalase from *E. coli*; αTA, branch chain amino acid transaminase from *E. coli*. GluDH, glutamate dehydrogenase from *E. coli* (Wu et al., 2016). **(C)** Production of different D-PheGly-derivatives starting from α-hydroxy-acids. MR, mandelate racemase; SMDH, (*S*)-mandelate dehydrogenase; DpgTA, D-PheGly aminotransferase; GluDH, glutamate dehydrogenase (Zhou et al., 2017). **(D)** Production of different D-PheGly-derivatives starting from styrenes. SMO, styrene monooxygenase; SpEH, epoxide hydrolase; AlkJ, alcohol dehydrogenase; ALDH, phenylacetaldehyde dehydrogenase; SMDH, (*S*)-mandelate dehydrogenase; DpgTA, D-PheGly aminotransferase. **(E)** Production of D-PheGly starting from L-Phe. Expansion of the previous system starting from styrenes was used, by addition of two additional enzymes. PAL: phenylalanine ammonia lyase; PAD, phenylacrylic acid decarboxylase (Zhou et al., 2017). **(F)** One-Pot biocatalytic synthesis of D-Tryptophan derivatives from indoles coupling tryptophan synthase (TrpS), LAAD and engineered D-alanine TA (engDATA; Parmeggiani et al., 2019b).

Natural pathway engineering has allowed the production of PheGly derivatives. As way of example, D-PheGly was produced in recombinant *E. coli* cells using phenylpyruvate as substrate, by a three-step route composed of hydroxymandelate synthase (HmaS), hydroxymandelate oxidase (HmO) and a stereoinverting hydroxyphenylglycine TA (HpgAT) (Figure 10E, Müller et al., 2006). The same strategy was used to produce L-PheGly, but using L-4-hydroxyphenylglycine transaminases from *A. orientalis* and *S. coelicolor* (Liu et al., 2014). Another synthetic biology-derived D-Phg operon has been recently developed on the basis of the natural *lpg* operon from *S. pristinaespiralis;* substitution of the natural PglE TA by the stereoinverting HpgAT allowed the creation of different plasmids which were utilized for the synthesis of D-PheGly in different engineered actinomycetal expression strains (Figure 11A, Moosmann et al., 2020).

Wu et al. reported different complementary enzymatic modules for one-pot conversion of styrenes to the corresponding (*S*)-α-hydroxy acids, (*S*)-amino alcohols, and (*S*)-α-amino acids in high yields and e.e. (>98%, Wu et al., 2016). Combination of 3 out of the 4 modules proposed in *E. coli* (Module 1: epoxidase and epoxide hydrolase; Module 2: alcohol dehydrogenase and aldehyde dehydrogenase. Module 4, hydroxy acid oxidase, L-enantioselective α-TA, catalase and glutamate dehydrogenase) allows for the production of different L-PheGly derivatives (Figure 11B). Besides the production of these compounds, this work represent a valid proof of concept of elegantly tackled multimodular whole cell factories, which is a general idea to construct different MECs (Farnberger et al., 2017; France et al., 2017). The previous modular strategy (Figure 11B, Wu et al., 2016) was further expanded for the enantioselective synthesis of D-PheGly derivatives starting from racemic mandelic acids, styrenes, or L-Phenylalanine derivatives. Firstly, in an analogous MEC to that presented previously (Figure 6D, Resch et al., 2010), mandelate racemase (MR), (*S*)-mandelate dehydrogenase (SMDH), D-PheGly aminotransferase (DpgAT) and GluDH built a MEC module to produce different D-PheGly derivatives, starting from different mandelic acids (Figure 11C); this was possible thanks to the unique stereoinverting L- to D-activity of DpgAT (Zhou et al., 2017). Conversions ranging 58–94% were achieved, with e.e. values ranging 93–98%. This module was adapted by removal of the MR and joined to two of those presented previously (modules 1 and 2, Figure 11B), in order to obtain D-PheGly derivatives starting from styrenes (Figure 11D). Finally, this MEC was further extended by inclusion of another module converting L-phenylalanine into styrene, by inclusion of PAL and phenylacrylic acid decarboxylase (PAD), turning into a MEC using nine different enzymes (Figure 11E, Zhou et al., 2017). D-PheGly was produced with this 8-step cascade, demonstrating they high efficiency of a long non-natural enzyme cascade/pathway.

Different D-Trp derivatives have been produced from substituted indoles coupling a tryptophan synthase from *Salmonella enterica*, LAAD from *Proteus* species, together with and an engineered D-alanine TA, with e.e. values ranging from 94 to >99% (Figure 11F, Parmeggiani et al., 2019b). One example of expansion of TA-based MECs toward the production of functionalized AA derivatives is provided by combination of an acetaldehyde-dependent aldolase (hydroxy-3-methyl-2-keto-pentanoate aldolase, HPAL) from *Arthrobacter simplex* together a branched-chain TA from *Bacillus subtilis* (BCAT). Aldol condensation of α-ketobutyrate and acetaldehyde followed by transamination allowed enzymatic synthesis of 4-hydroxy-L-isoleucine (4HIL) (Smirnov et al., 2007). Whereas the final yield of 4HIL was not high, the authors proposed that this problem might be overcome by shifting the equilibrium of the reaction by separation of the reaction products and recirculation of the unreacted substrates.

### Other MECs for the Synthesis of NcAAs

Other multienzymatic or chemoenzymatic strategies have been proposed for the synthesis of NcAAs, although they have not found yet such a biotechnological interest as those reported above. On the other hand, it is important to bear in mind that besides the more than 20 different enzymes comprised in the different MECs presented in this paper, many enantioselective or stereospecific “free” enzymes are useful for NcAAs synthesis [e.g., proteases (Nakao et al., 1996; Miyazawa, 1999), 2-oxoglutarate-dependent oxygenases (Peters and Buller, 2019), aldolases (Xue et al., 2018; Fesko, 2019) or other different PLP-enzymes (Di Salvo et al., 2020)].

#### Lipase-Containing MECs

**FIGURE 12:**
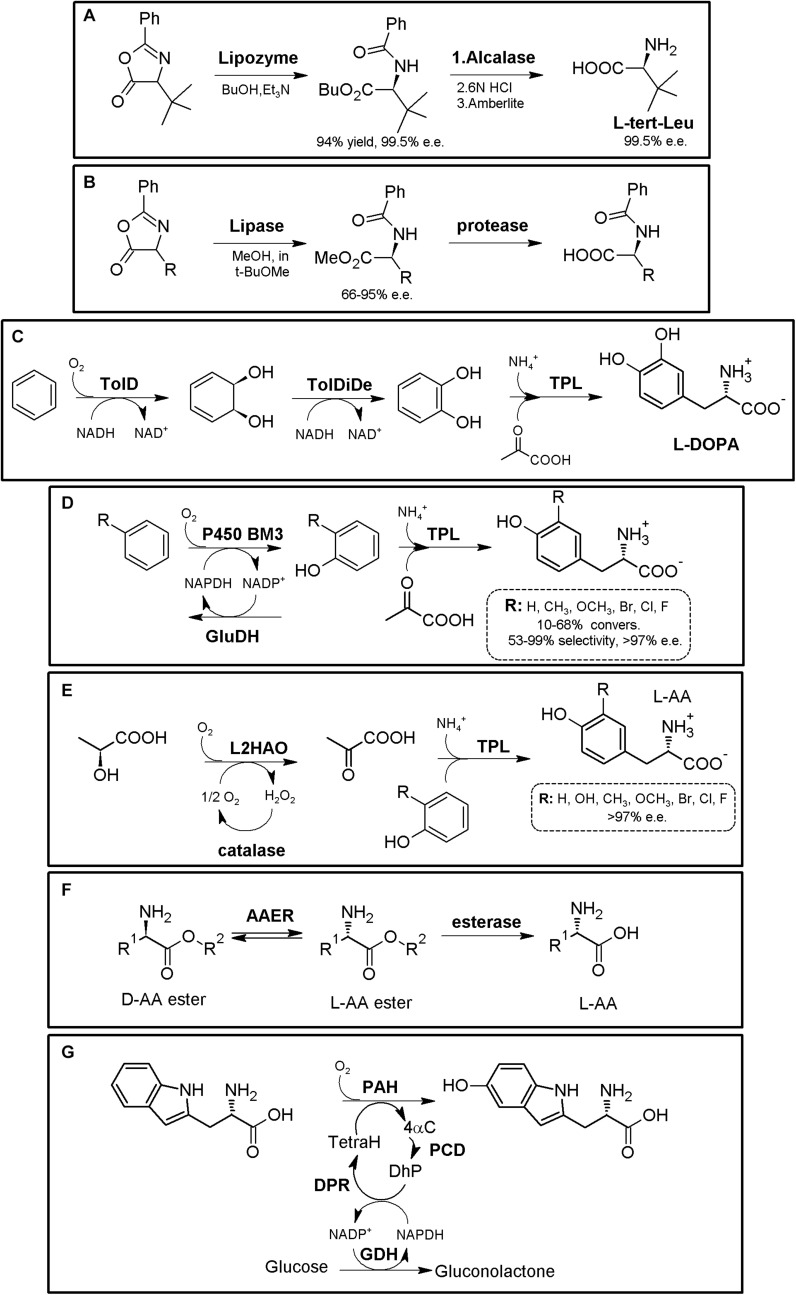
Other MECs for the synthesis of NcAAs. **(A)** Production of L-*tert*-Leu using Lipozyme and Alcalase, followed by chemical hydrolysis. Treatment of racemic 2-phenyl-4-*tert*-butyloxazolin-5(4*H*)-one in toluene containing *n*-butanol with lypozyme and a catalytic amount of trimethylamine, resulted in ((*S*)-*N*-benzoyl-*tert*-leucine butyl ester (yield 94%). Two additional hydrolysis steps using Alcalase^®^ and 6N HCl, yielded enantiopure L-*tert-*leucine (Turner et al., 1995). **(B)** Proposed lipase/protease system for the hydrolysis of production of different enantio-enriched *N*-benzoyl-L-α-AAs (Miyazawa, 1999). **(C)** Production of L-DOPA by a hybrid pathway using toluene dioxygenase (TolD), toluene-*cis*-glycerol de-hydrogenase (TolDiDe) and Tyrosine phenol lyase (TPL) (Park et al., 1998; Min et al., 2015). **(D)** Production of different L-Tyr derivatives using P450-BM3 with a NADPH regeneration system and engineered TPL (Dennig et al., 2015; Parmeggiani et al., 2018). **(E)** Transformation of L-lactate and o-phenols into enantiopure L-Tyr derivatives. L2HAO: L-2-hydroxy acid oxidase (e.g., AVLOX from *Aerococcus viridans*); TPL: WT or engineered tyrosine phenol lyase from *Symbiobacterium toebii* (Li et al., 2020). **(F)** Theoretical approach for the deracemization of AA ester racemates using an “amino acid ester racemase” (AAER, an ACLR homolog) and an enantioselective esterase (Frese et al., 2018). **(G)** 5-Hydroxy-tryptophan synthesis through a L-phenylalanine 4-hydroxylase coupled to cofactor regeneration. PAH, L-phenylalanine 4-hydroxylase; PCD, pterin-4α-carbinolamine dehydratase; DPR, dihydropteridine reductase; GDH, glucose dehydrogenase TetraH, tetrahydropterin; 4 α C, 4α-carbinolamine; DhP, dihydropteridine (Hara and Kino, 2013).)

Lipases are important enzymes from the industrial point of view, due to its huge versatility and robustness in the production of different compounds. Despite their outstanding promiscuous properties, one drawback that lipases (and proteases) can show is the lack of a perfect enantioselectivity, but several enantiopure compounds can be prepared using them. A bienzymatic method composed of Lipozyme^®^ (*Mucor miehei*) and Alcalase^®^ was applied for the production of enantiopure L-tert-leucine (99.5% e.e., Figure 12A) (Turner et al., 1995). A similar system was proposed starting from different 5(4H)-oxazolone derivatives (Figure 12B); *P. cepacia* lipase hydrolyzes different oxazolone derivatives yielding optically active *N*-benzoyl-L-α-AA methyl esters. Subsequent methyl ester hydrolysis by proteases (prozyme 6 and protease N; pH 6.8), might yield *N*-benzoyl-L-α-AAs of high enantiomeric purity (Figure 12B; Crich et al., 1993; Miyazawa, 1999).

A chemoenzymatic approach taking advantage of lipase and acylase I has been used for the production of enantiopure L-α-NcAAs starting from *N*-acetyl-α-AAs (Podea et al., 2008; Bencze et al., 2015). However, it is important to highlight that acylase I alone allows deracemization of these compounds, without the need of the lipase mediated DKR. Moderate yields (approximately 80%) of enantiopure L-benzofuranyl- and L-benzothienyl alanines were produced (e.e. > 99%; Podea et al., 2008). A similar strategy allowed the production of different enantiopure L-(5-phenylfuran-2-yl)alanines through a sequential multi-enzyme process based in lipase Cal-B, Pig liver esterase and Acylase I starting from racemic 2-acetamido-3-(5-phenylfuran-2-yl)propanoic acids. The target compounds were produced in 81–84% yield, with >99% e.e. (Bencze et al., 2015).

#### Tyrosine Phenol Lyase-Containing MECs

Tyrosine phenol lyase (TPL, β-tyrosinase) has been applied on different MECs for the production of interesting pharmaceutical compounds, including the enantioselective synthesis of L-Phe/L-Tyr derivatives (Martínez-Montero et al., 2019). Coupling with DAAO was proposed three decades ago (Figure 4B, section “Amino Acid Oxidase-Based MECs”). Park et al. (1998) designed a hybrid pathway using toluene dioxygenase, toluene *cis*-glycerol de-hydrogenase, and TPL for the production of L-DOPA using benzene as substrate (Figure 12C). This strategy presented toxicity drawbacks since it was used in whole-cell systems (Park et al., 1998). A somehow similar system obtained by coupling of monooxygenase P450-BM3 with a NADPH regeneration system an engineered TPL allowed one-pot synthesis of L-Tyr derivatives starting from monosubstituted benzenes, pyruvate, and ammonia (Figure 12D, Dennig et al., 2015). L-lactate oxidases coupled to a catalase and a designed thermophilic TPL (TTPL) was applied for one-pot synthesis of L-tyrosine derivatives (Figure 12E, Li et al., 2020). The best lactate oxidase studied (AvLOX) was used *for in situ* generation of pyruvate starting from L-lactate (also obtained with another enzyme cascade, and thus, they might be coupled); different o-phenol acceptors were used for TTPL. Using 28–36 h of reaction, recovery yields ranging 51–84% were obtained for the different L-Tyr derivatives (Li et al., 2020).

#### Tryptophan Synthase-Containing MECs

Tryptophan synthase (TrpS) has proved an interesting tool of expansion of NcAA MECs, since it has been engineered to accept a wide range of compounds, such as nitroalkanes, nitroindoles or 3-substituted oxindoles for the production of many different NcAAs (Buller et al., 2015; Herger et al., 2016; Romney et al., 2017, 2019; Boville et al., 2018a, b), leading even to new quaternary stereocenters (Dick et al., 2019). TrpS has also been engineered to accept L-Thr as a substrate for the synthesis of β-methyltryptophan derivatives, which could be further halogenated by a halogenase (Francis et al., 2017).

TrpSs have been included in AAO- and TA-based MECs (sections “Amino Acid Oxidase-Based MECs” and “Transaminase-Based MECs”; Parmeggiani et al., 2019b; Schnepel et al., 2019). On the other hand, a series of NcAAs including L-Trp and L-Cys derivatives were synthesized using a D-threonine aldolase/TrpS/Alanine Racemase system in high conversion and excellent enantioselectivity (e.e. > 99%) starting from Gly and paraformaldehyde. In a first step, D-threonine aldolase from *Arthrobacter* sp transformed the substrates into D-serine with high yield (240 g/L). Racemization into D,L-Ser was carried out with alanine racemase from *Bacillus subtilis*; L-ser was then transformed into different L-NcAAs by coupling with various indoles/thiols using L-tryptophan synthase from *E. coli* (Yu et al., 2019).

#### AAER/Esterase System

Recently, the deracemization of AA esters through the action of a combination of an AA-ester racemase (AAER) and an enantioselective esterase has been proposed (Frese et al., 2018). Different PLP-dependent ACLR-homologs (see section “Amidase Process”) with AAER activity on phenylalanineamide were identified, thus allowing to propose a putative AAER/esterase system for deracemization of AA esters, analogous to the “Amidase Process” (Figure 12F).

#### Other MECs Taking Advantage of Metabolic Pathways

Besides some of the examples shown in this work, many additional metabolic natural or engineered MECs exist in the literature. A recent seminal and must read review on biosynthetic pathways to NcAAs has been published (Hedges and Ryan, 2020), and the reader is referred to it for further information on these systems. As isolated examples, the L-Trp biosynthesis pathway from *E. coli* has been expanded by inclusion of engineered L-Phenylalanine 4-hydroxylase from *C. violaceum* (Figure 12G, Hara and Kino, 2013) or engineered aromatic amino acid hydroxylase from *Cupriavidus taiwanensis* (Mora-Villalobos and Zeng, 2018); whole cell systems for 5-HTP depend on the elimination of the *E. coli* tryptophanase gene, and inclusion of recycling of cofactors. Approx. 2–4 mM 5-HTP was produced using those systems. Natural routes for PheGly derivative synthesis have been reported, which might be further exploited for future MEC development (Al Toma et al., 2015). In this sense, special consideration should be paid to natural secondary metabolic routes conducting to NcAAs (or using them) to expand established MECs for their synthesis. Metabolic engineering of microorganisms have also allowed production of NcAAs such as L-norvaline/L-norleucine (Anderhuber et al., 2016), L-citruline (Eberhardt et al., 2014), hydroxyproline (Falcioni et al., 2015), L-ornithine (Wu et al., 2020) different D-α-AAs (Mutaguchi et al., 2018) or different *N*-Alkylated AAs (Mindt et al., 2019, 2020; van der Hoek et al., 2019). Thus, isolated enzymes comprised in biosynthetic metabolic routes for the production of different compounds (Hedges and Ryan, 2020) might find application on some of the existing MECs described in this paper.

## Conclusion

In this review, we have brought together different established MECs and other less-used approaches for the biosynthesis of several NcAAs, providing a general overview on different methodologies available in the literature. Some of the enzymes described in this review have also been evolved to alter their substrate scope, widening their application, and inviting to revisit the old methodologies for further development. Enzymes from biosynthetic pathways to NcAAs (Hedges and Ryan, 2020) are clear candidates for MECs expansion. On the other hand, information of specific enzymes for functionalization of AAs or AA transformation into other important pharmaceutical compounds is also accumulating in the literature (e.g., Smirnov et al., 2012; Busto et al., 2014; Hönig et al., 2017; Hyslop et al., 2018; Parthasarathy et al., 2018; Peters and Buller, 2019; McDonald et al., 2019; Hedges and Ryan, 2020; Mindt et al., 2020; Song et al., 2020; Wendisch, 2020; Zhang et al., 2020; Zhao et al., 2020). These enzymes are, in principle, complementary to well stablished NcAA-production MECs. As way of example, promiscuous L-tryptophan decarboxylases have been applied for the synthesis of different triptamines (McDonald et al., 2019), and might be coupled to TprS-containing MECs for their expansion. Different proteases have also been applied to produce amino acid-ester derivatives starting from amino acids (Song et al., 2020), which might be coupled to several MECs proposed in this review. Thus, whereas the discovery of new or newly designed enzymes continues being of great interest, a “*back and to the future*” strategy might also speed up the “Fourth Wave of Biocatalysis” by dusting off previous enzymatic methodologies. We expect that potential readers in the field find the information contained in this paper helpful to speed up the generation of new or improved MECs.

## Author Contributions

SM-R designed and directed the idea of this review. All the authors participated in literature search, writing, and comments on the manuscript.

## Conflict of Interest

The authors declare that the research was conducted in the absence of any commercial or financial relationships that could be construed as a potential conflict of interest.

## Footnotes

1 ω-TAs are also valuable for the synthesis of chiral amines to their broad substrate spectrum, and do not necessarily precise the presence of the carboxylate group in the reactant.

2 A historical perspective on this process can be consulted in Martínez-Rodríguez et al. (2020).

3 This enzyme belongs to the ammonia-lyase family; L-threonine ammonia lyase, ThrD, E.C. 4.3.1.19), and is not a “real” L-amino acid deaminase.

4 Since (i) reduction of the e.e. value of the produced amino acid was observed over time, (ii) the reaction conditions used pHs above 9, and (iii) chemical racemization of AAs is known to occur at high pHs, the formation of the opposite enantiomer might be the result of chemical racemization.

5 The nomenclature of TAs in the literature can be confusing. We recommend reading of the new classification carried out by Cutlan et al. (2019).

6 Inclusion of a LAAO yields an analogous MEC from the theoretical point of view.
