# Supplementary material for: Overview on Multienzymatic Cascades for the Production of Non-canonical α-Amino Acids
Source: Front Bioeng Biotechnol. 2020 Aug 11;8:887. doi: 10.3389/fbioe.2020.00887 (PMC7431475; doi:10.3389/fbioe.2020.00887)
Supplement: Supplementary file 1 [file Table_1.DOCX]

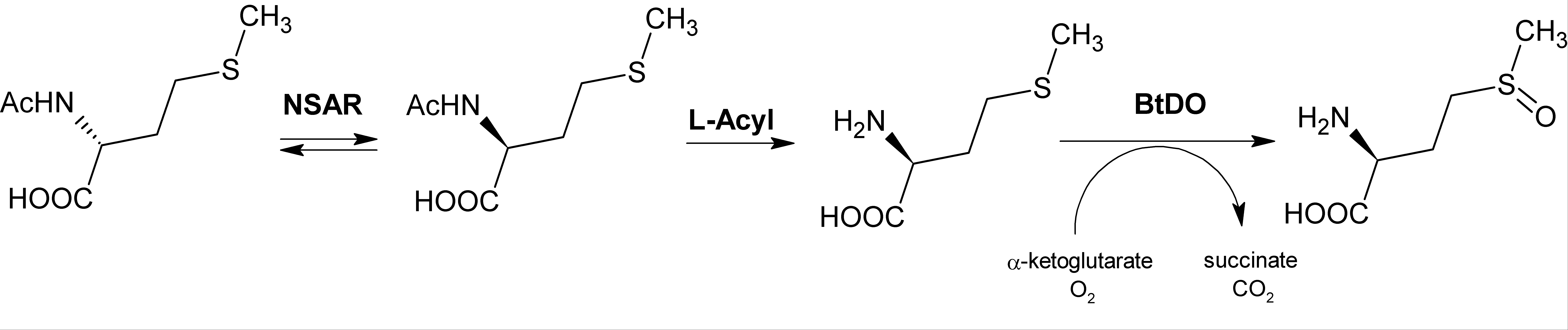


Figure 1SM. Stereoselective production of γ-oxyfunctionalyzed AAs. Engineered NSAR mutant (G291D/F323Y) from *Amycolatopsis* sp. Ts1-60 (NSAR); L-aminoacylase from *Geobacillus thermoglucosidasius (*L-Acyl). BtDO: stereoselective isoleucine dioxygenase from *Bacillus thuringiensis.* L-methionine-(*S*)-sulfoxide with 97% yield and 95% d.e. was produced (Enoki et al, 2016).





Figure 2SM. Preparation of L-α-ABA starting from L-Thr using Threonine deaminase (ThrD), aromatic aminotransferase (TyrTA), and acetolactate synthase (AceS), together alanine racemase (AlaR)/D-amino acid oxidase (DAOO) for removal of the by product L-Ala produced during the reaction (Zhu et al, 2011). The AceS pyruvate-degrading pathway and the AlaR/DAOO pyruvate-recycling route are marked by dashed lines.





Figure 3SM. One-pot MEC reaction for the production of D- and L‐β‐methyl‐α‐AAs using enantioselective SAM‐dependent α‐keto acid methyltransferases (MT), an halide methyltransferase (HMT) and a D- or L-α‐aminotransferase (D- or L-TA) cooperating in two orthogonal reaction cycles (Liao and Seebeck, 2020).





Figure 4SM. A) Enzymatic synthesis of [2-^3^H]-L-Tyr starting from chemically synthesised [2-^3^H]-cinnamic acid using L-phenylalanine ammonia lyase and L-Phe-4'-monooxygenase from rat liver (4MO, EC 1.14.16.1; Pajak et al, 2018). The reaction was carried out in the presence of D,L-6-methyl-5,6,7,8-tetrahydropterine (THP, 4MO cofactor) and D,L-dithiothreitol. B) Theoretical production of L-DOPA coupling the previous system to p-hydroxyphenylacetate 3-hydroxylase (PHAH, Min et al, 2015), also including THP- (Hara and Kino, 2013) and NADH-recycling systems

**References**

Enoki, J., Meisborn, J., Müller, A.C., Kourist, R. (2016). A Multi-Enzymatic Cascade Reaction for the Stereoselective Production of γ-Oxyfunctionalyzed Amino Acids. Front. Microbiol. 7:425. doi: 10.3389/fmicb.2016.00425

Hara, R., and Kino K. (2013). Enhanced synthesis of 5-hydroxy-l-tryptophan through tetrahydropterin regeneration. AMB Express 3:70. doi:10.1186/2191-0855-3-70

Liao, C., and Seebeck F.P. (2020). Asymmetric β-methylation of L- and D-α-amino acids by a self-contained enzyme cascade. Angew. Chem. Int. Ed. 59, 7184-7187.

Pająk, M., Pałka, K., Winnicka, E., Kańska, M. (2018). The chemo-enzymatic synthesis of labeled l-amino acids and some of their derivatives. J. Radioanal. Nucl. Chem. 317, 643-666.

Zhu, L., Tao, R., Wang, Y., Jiang, Y., Lin, X., Yang, Y., et al. (2011). Removal of L-alanine from the production of L-2-aminobutyric acid by introduction of alanine racemase and D-amino acid oxidase. Appl. Microbiol. Biotechnol. 90, 903-910.
